# The impact of food reformulation on nutrient intakes and health, a systematic review of modelling studies

**DOI:** 10.1186/s40795-018-0263-6

**Published:** 2019-01-07

**Authors:** Carlo Federici, Patrick Detzel, Francesco Petracca, Livia Dainelli, Giovanni Fattore

**Affiliations:** 1 CeRGAS (Centre for Research on Health and Social Care Management), SDA Bocconi School of Management, Milan, Italy; 2 Nestlé Research Center, Nestec SA, Lausanne, Switzerland; 3 Department of Policy Analysis and Public Management, Bocconi University, Milan, Italy

**Keywords:** Food reformulation, Modelling, Nutrition policies, Public health, Decision-analytical models, Sodium intake, Sugar intake, Fat intake

## Abstract

**Background:**

Unhealthy diet is a risk factor for adverse health outcomes. Reformulation of processed foods has the potential to improve population diet, but evidence of its impact is limited. The purpose of this review was to explore the impact of reformulation on nutrient intakes, health outcomes and quality of life; and to evaluate the quality of modelling studies on reformulation interventions.

**Methods:**

A systematic review of peer-reviewed articles published between January 2000 and December 2017 was performed using MEDLINE, ScienceDirect, Embase, Scopus, Cochrane, and the Centre for Reviews and Dissemination of the University of York. Additional studies were identified through informal searches on Google and specialized websites. Only simulation studies modelling the impact of food reformulation on nutrient intakes and health outcomes were included. Included articles were independently extracted by 2 reviewers using a standardized, pre-piloted data form, including a self-developed tool to assess study quality.

**Results:**

A total of 33 studies met the selected inclusion criteria, with 20, 5 and 3 studies addressing sodium, sugar and fats reformulation respectively, and 5 studies addressing multiple nutrients. Evidence on the positive effects of reformulation on consumption and health was stronger for sodium interventions, less conclusive for sugar and fats. Study features were highly heterogeneous including differences in methods, the type of policy implemented, the extent of the reformulation, and the spectrum of targeted foods and nutrients. Nonetheless, partial between-study comparisons show a consistent relationship between percentages reformulated and reductions in individual consumption. Positive results are also shown for health outcomes and quality of life measures, although comparisons across studies are limited by the heterogeneity in model features and reporting. Study quality was often compromised by short time-horizons, disregard of uncertainty and time dependencies, and lack of model validation.

**Conclusions:**

Reformulation models highlight relevant improvements in diets and population health. While models are valuable tools to evaluate reformulation interventions, comparisons are limited by non-homogeneous designs and assumptions. The use of validated models and extensive scenario analyses would improve models’ credibility, providing useful insights for policy-makers.

**Review Registration:**

A research protocol was registered within the PROSPERO database (ID number CRD42017057341).

**Electronic supplementary material:**

The online version of this article (10.1186/s40795-018-0263-6) contains supplementary material, which is available to authorized users.

## Background

The growing evidence on the detrimental effects of poor diet on health, including cardiovascular diseases, diabetes and some type of cancers [1–5], has stimulated national and international nutrition programs and policies to strive to reduce the intake of sodium, sugar and fat to the recommended limits [6, 7]. Such initiatives largely focus on changing individuals’ behavior by promoting social marketing campaigns, community-based interventions and/or primary healthcare programs, whereas other possible strategies target the dietary environment by affecting availability and prices of unhealthy foods. More recently, increasing attention has been given to interventions favoring the reformulation of processed foods towards healthier alternatives [8, 9]. Reformulation is defined as the process of altering a food or beverage product’s recipe or composition to improve the product’s health profile [10]. These strategies are consistent with the World Health Assembly 2004 report that called for initiatives by the food industry to reduce the content of harmful nutrients in processed foods and increase the introduction of innovative, healthy and nutritious choices. However, the current policy agenda of reformulation strategies exhibits a prevailing focus on the former objective, thus aiming to remove or reduce public health sensitive nutrients from foods, while maintaining the same appearance, use and characteristics such as flavor, texture and shelf life [11]. According to a recent review, 59 out of 83 countries have on-going programs of work with the industry to reduce sodium in processed foods [12]. To a minor extent, similar efforts are now in place to reduce excess intakes of sugar, particularly from sugar sweetened beverages, and fat [13].

However, despite the growing emphasis given to reformulation, evidence of its impact on both nutrient intakes and health outcomes is limited. Several design characteristics may affect the policy impact. These include i) the voluntary or mandatory nature of the program, and its consequences on the adherence from the industry; ii) the breadth of the reformulation in terms of products targeted; iii) the amount and type of nutrients reformulated; and iv) the pace of implementation, including how reduction targets are set scheduled and reached.

Additionally, how consumers might react to reformulated products and how this will affect the overall policy effectiveness remains largely unexplored, especially for sugar and fat reformulations. Indeed, consumers might react to changes in taste and energy density by shifting to other, non-reformulated products, eating more, or increasing the use of additional ingredients (e.g. discretionary salt and sugar).

Due to these features, estimating the effectiveness of current and future reformulation policies is challenging, and likely to be both policy and context specific.

Given to the obvious difficulties of performing experimental designs to measure the impact of nutrition policies on population health [14], the majority of published studies use mathematical models to predict the effects of reformulation on intakes and clinical outcomes [15]. Nonetheless, the extreme flexibility of modelling, and the required assumptions needed to simplify complex nutrition interventions may introduce a considerable variability, thus limiting between-study comparability and even challenging the plausibility of models results for population health [16]. To the authors’ best knowledge, only one previous review collected and critically appraised analytical models predicting the effects of several nutrition interventions, including reformulation, on intake and health [14]. In the study, reformulation was found to have positive effects on intake and health outcomes; however, due to the broader scope of the authors’ work, detailed considerations of reformulation-specific characteristics were only partially addressed.

Therefore, the objectives of this review are to focus on reformulation studies aimed at reducing the content of harmful nutrients to: i) further explore the impact of reformulation on the intake of target nutrients, as well as on health outcomes and quality of life measures; and ii) critically evaluate the quality of reformulation models, with a focus on the key elements that are specifically relevant to their appraisal.

## Methods

A research protocol was previously defined following the PRISMA-P guidelines [17, 18], and registered within the PROSPERO database (ID number CRD42017057341). Studies were reviewed according to the PRISMA guidelines [19] (Additional file 1).

### Search strategy

Records published between January 2000 and December 2017 were searched in electronic bibliographic databases including Medline (via Web of Science), ScienceDirect, Embase, Scopus, the Cochrane and Cochrane Public Health Group Specialized Register, and the Centre for Reviews and Dissemination of the University of York. The full search strategy was first defined on Web of Science (Additional file 2), and then adapted to the other databases. Grey literature was retrieved through informal searches on Google, and on the following websites: Opengrey (http://www.opengrey.eu/), WHO, CDC, and the FAO websites.

### Selection of studies

Studies were considered eligible if they addressed mandatory or voluntary food reformulation strategies aimed at reducing individual intake of sodium, saturated fatty acids (SFA), trans-fatty acids (TFA), and sugar. For studies addressing sugar intake, all definitions were accepted, including added sugars, total sugars and free sugars. Included reformulation strategies were required to be aimed at healthy individuals without any age restriction, and to target foods commonly available in retail stores. On the contrary, initiatives limited to restaurants or specific settings such as hospitals, schools, and workplace, or interventions targeting fortification and improved intake of “healthy” nutrients were considered out of scope.

The primary outcomes of interest were i) changes in individual intakes of target nutrients; ii) effects on health outcomes, including obesity, incidence of cardiovascular diseases (CVD), type 2 diabetes (T2D), life-years gained or reduced mortality; and iii) changes in health-related quality of life measures, i.e. Quality Adjusted Life Years (QALYs) and Disability Adjusted Life Years (DALYs).

Although the types of studies to be included were not set in advance in the research protocol, a focus was later given on mathematical and statistical models, since a first scoping of the literature showed that modelling studies were by far the most frequent study design to assess the effects of reformulation policies on intake and health outcomes. Focusing on model-specific issues for reformulation interventions was then deemed more relevant than reporting results from a broader set of study designs.

Cost-effectiveness models were included as well. However, cost data were not reported, since such policies are usually cost-saving [20], and the main focus of the present review was to assess the magnitude of the effects on intake and health outcomes, rather than cost-effectiveness. Finally, only studies in English were considered for review.

### Data extraction

One reviewer (CF) defined the search strategy and imported the retrieved records in Endnote (ver. X6), whereas two reviewers (CF and FP) independently performed title and abstract screening, full-text analysis and data extraction. At each stage, discrepancies were resolved by discussion.

A standardized, pre-piloted form was used for data extraction including data on: model type; target population; time horizon of the analysis; target country; mandatory/voluntary nature of the policy; target nutrients; data sources used in the models and funding sources.

Even if not explicitly stated, interventions where all products consumed/marketed are reformulated were assumed to be likewise mandatory interventions, as it was assumed that no voluntary policy would achieve such degree of pervasiveness across manufacturers.

The effects of the interventions were collected and reported for each causal step from reduction in intake, through its effects on risk factors levels (e.g. blood pressure), to the impact on clinical outcomes and health related quality of life measures. When reported, both absolute and percentage reductions were collected. To improve between-study comparability, estimates of QALYs or DALYs were reported per 100,000 individuals whenever the total size of the population was reported by the original studies. In addition, salt amounts were converted to sodium amounts using a 1 g/400 mg conversion rate.

In addition, when available, further data were extracted on the technical feasibility of the modelled interventions, and on whether potential behavioral changes among consumers had been considered in the studies.

If modelling results were provided separately by population sub-groups (e.g. age or sex), the weighted mean was calculated across estimates.

### Quality assessment of studies

There are no specific tools to assess the quality of modelling studies evaluating population-wide nutrition interventions. Therefore, a self-developed evaluation tool was used, mainly drawing from relevant criteria recommended by the International Society for Pharmacoeconomics and Outcomes Research (ISPOR) Good Practice Task Force [21]. The proposed tool identifies 9 criteria to assess both the scientific quality of the studies (model validation, credible data inputs, uncertainty analysis, transparency and reporting quality), and the pertinence to the research question (objective, scope and relevance). The full list of criteria and the evaluation guide are provided in the Additional file 3.

## Results

**Fig. 1:**
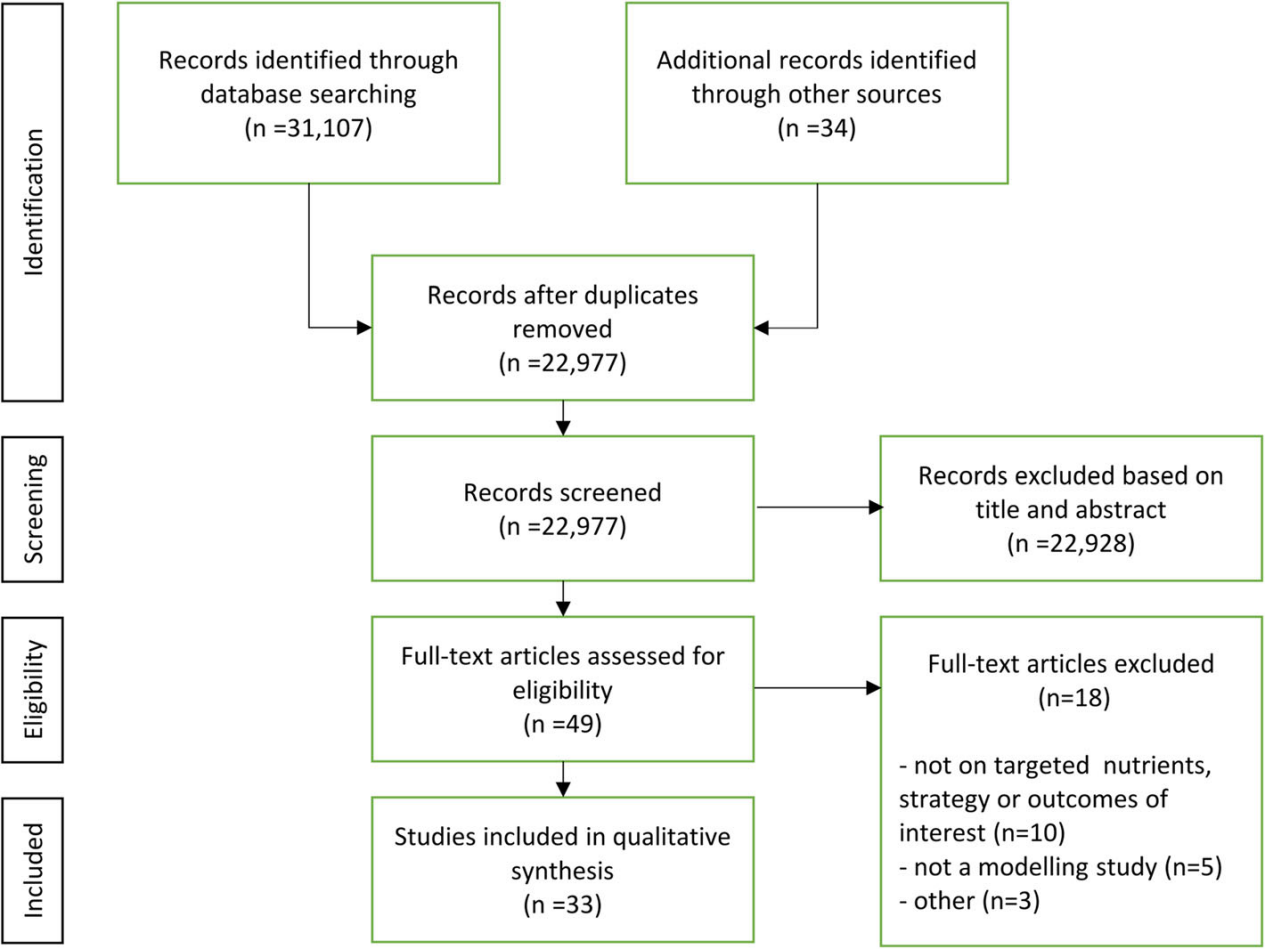
flow chart of the literature search process

After removing duplicates, the literature search identified 22,907 records from bibliographic databases and other sources. After abstract and title screening, 49 records were analyzed full-text and 33 studies were finally included in the review. Reasons for full-text exclusion were documented and are reported in the flow diagram (Fig. 1).

### Study characteristics

**Fig. 2:**
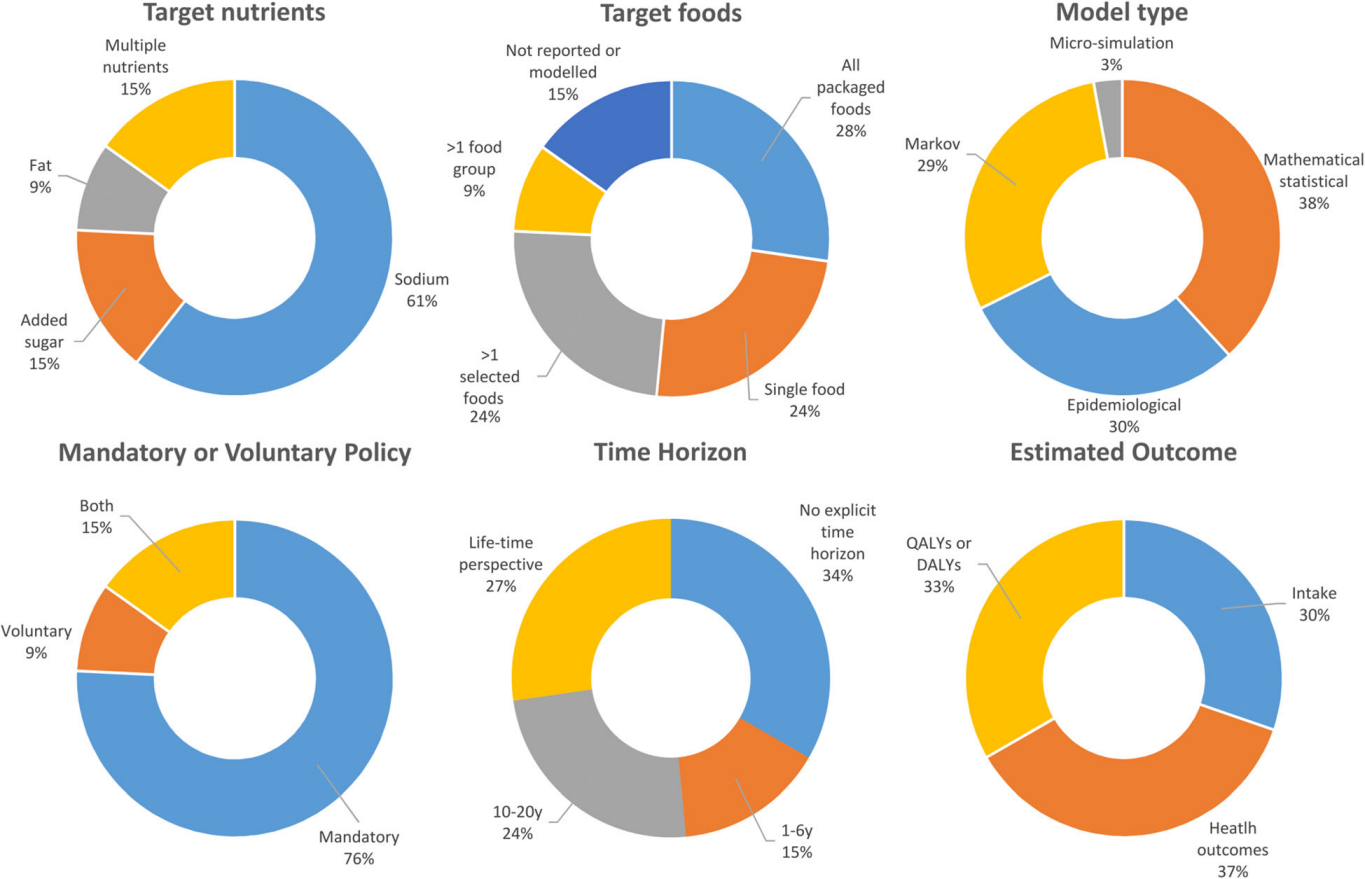
Characteristics of the included studies

Tables 1, 2 and 3 Fig. 2 and the Additional file 4 summarize the main findings and characteristics of the included studies. Overall, studies were heterogeneous in the way interventions and outcomes were modelled and reported, limiting between-study comparisons. As regards the type of nutrient reformulated, 20 studies focused exclusively on sodium reduction (60.6%) [22–41], followed by 5 studies considering at least 2 different nutrients (15.1%) [8, 42–45], 5 studies addressing sugar (15.1%) [46–50] and 3 study addressing fat (9%) [11, 51, 52].

**Table 1:** Interventions targeting sodium consumption

Author (year)	Study Characteristics					Study Outcomes			
	Model type	Time horizon	Target foods	Type of intervention(s)	Voluntary or mandatory	Reduction in intake	Reduction in blood pressure (BP)	Life years gained and reduction in the incidence/prevalence of health outcomes	Results on QOL measures
Cogswell et al. (2017) [29]	Mathematical/Statistical	Not modelled	All processed food	↓28% in Na content (Health Canada Benchmarks)	Mandatory	0.61 Na g/day (22%, UI = 0.59–0.63)	–	–	–
Choi et al. (2016) [30]	Micro-simulation	10y	All processed foods	Product reformulated to meet product-specific NSRI criteria extended to all food producers in the US	Mandatory	0.365 (SE = 0.9) Na g/day (10.9%)	–	Hypertension: 0.97%	–
								All AMI: 2.59%	
								All strokes: 2.67%	
								Fatal AMI: 0.36%	
								Fatal Strokes: 0.23%	
Food and Drink Industry Ireland (2016) [26]	Mathematical/Statistical	Not modelled	10 Food macrocategories	Reformulation based on actual FDII voluntary programme	Mandatory extension of existing programme	0.57 Na g/day (17.8%) in adults	–	–	–
					Voluntary	0.06 Na g/day (2.3%) in adults	–	–	–
Leroy et al. (2016) [32]	Epidemiological	1y	F&V, bread, meat, fish, sandwiches, sauces	Strong reformulation based on the Choices Programme criteria	Mandatory	12.7% daily Na intake	–	Fatal CVD/Strokes deaths averted: 422	–
								Cancer deaths averted: 187	
				Mild reformulation based on the Choices Programme criteria	Mandatory	9.3% daily Na intake	–	CVD/Strokes and cancer deaths averted: 2408 (3.7%) - due to total reductions in Na, SFA and sugar consumption combined	–
Masset et al. (2016) [25]	Mathematical/Statistical	Not modelled	Pizza	Reformulation to meet Nestlè Nutrient Profiling targets	Mandatory	0.14 Na g/day (4%)	–	–	–
Nghiem et al. (2016) [42]	Markov	Cohort life-time	All processed foods	59% substitution of NaCl with other salts (K, Mg)	Mandatory	1.82 Na g/day (51.5%)	–	–	12,783 QALYs gained/100000 pop. (UI = 10,348–15,609)
				25% substitution of NaCl with other salts (K, Mg)	Mandatory	0.77 Na g/day (21.8%)	–	–	5261 QALYs gained/100000 pop. (UI = 4230–6391)
			Bread	↓38,5% in NaCl content	Mandatory	0.28 Na g/day (7.9%)	–	–	1891 QALYs gained/100000 pop. (UI = 1509–2296)
				↓11,1% in NaCl content	Mandatory	0.08 Na g/day (2.3%)	–	–	678 QALYs gained/100000 pop. (UI = 548–822)
Wilson et al. (2016) [43]	Markov	Cohort life-time	All processed foods (bread, processed meats, sauces, snack food, bakery, cheese)	↓36% in NaCl content across product types	Mandatory	0.628 Na g/day	–	–	5304 QALYs gained/100000 pop. (UI = 4270–6478)
					Voluntary	Same efficacy with higher uncertainty	–	–	5000 QALYs gained/100000 pop. (UI = 3709–6391)
			Bread	↓12–37% in NaCl content across bread types	Mandatory	0.043 Na g/day	–	–	387 QALYs gained/100000 pop.(UI = 309–470)
					Voluntary	Same efficacy with higher uncertainty	–	–	365 QALYs gained/100000 pop. (UI = 270–461)
			Processed meats	↓35–55% in NaCl content overall	Mandatory	0.069 Na g/day	–	–	583 QALYs gained/100000 pop. (UI = 470–704)
					Voluntary	Same efficacy with higher uncertainty	–	–	552 QALYs gained/100000 pop. (UI = 417–696)
			Sauces	↓30–63% in NaCl content across sauces types	Mandatory	0.104 Na g/day	–	–	870 QALYs gained/100000 pop. (UI = 700–1057)
					Voluntary	Same efficacy with higher uncertainty	–	–	822 QALYs gained/100000 pop. (UI = 626–1039)
			Combination of bread, processed meats and sauces	–	Mandatory	0.217 Na g/day	–	–	1843 QALYs gained/100000 pop. (UI = 1487–2239)
					Voluntary	Same efficacy with higher uncertainty	–	–	1743 QALYs gained/100000 pop. (UI = 1326–2204)
			Snack food	↓34–48% in NaCl content across snacks types	Mandatory	0.032 Na g/day	–	–	265 QALYs gained/100000 pop. (UI = 217–322)
					Voluntary	Same efficacy with higher uncertainty	–	–	252 QALYs gained/100000 pop. (UI = 191–317)
			Bread and bakery products	↓12–37% in NaCl content across bread types; ↓54–63% in NaCl content across other bakery products	Mandatory	0.107 Na g/day	–	–	887 QALYs gained/100000 pop. (UI = 722–1078)
					Voluntary	Same efficacy with higher uncertainty	–	–	843 QALYs gained/100000 pop. (UI = 639–1061)
			Cheese	↓27–42% in NaCl content across cheese types	Mandatory	0.045 Na g/day	–	–	383 QALYs gained/100000 pop. (UI = 309–461)
					Voluntary	Same efficacy with higher uncertainty	–	–	361 QALYs gained/100000 pop. (UI = 274–457)
Bruins et al. (2015) [45]	Mathematical/Statistical	Cohort life-time	Soups	↓25% in Na content	Mandatory	0.05 Na g/day	0.11 mmHg	Strokes: 0.49%	6.45 DALYs averted/100000 pop
								AMI: 0.34%	
								Angina: 0.34%	
								CHF: 0.24%	
Dötsch-Klerk et al. (2015) [23]	Mathematical/Statistical	Not modelled	All processed foods	Products reformulated to meet the 6 g/day NaCl consumption target	Mandatory	US: 1.8 Na g/day (23%)	–	–	–
						UK: 1.8 Na g/day (27%)	–	–	–
						NL: 1.3 Na g/day (19%)	–	–	–
				Products reformulated to meet the 5 g/day NaCl consumption target	Mandatory	US: 2.2 Na g/day (28%)	–	–	–
						UK: 2.1 Na g/day (32%)	–	–	–
						NL: 1.8 Na g/day (26%)	–	–	–
Gillespie et al. (2015) [31]	Epidemiological	10y	All processed foods	↓30% in NaCl	Mandatory	0.58 Na g/day (UI = 0.56–0.60)	0.81 mmHg (UI = 0.53–1.10)	CHD deaths averted or postponed: 4467 (UI = 2854–6147)	–
				↓10% in NaCl	Mandatory	0.19 Na g/day (UI = 0.18–0.20)	0.27 mmHg (UI = 0.18–0.37)	CHD deaths averted or postponed: 1502 (UI = 953–2068)	–
				↓24% in NaCl	Voluntary (applied to 39% of products)	0.19 Na g/day (UI = 0.03–0.63)	0.27 mmHg (UI = 0.04–0.92)	CHD deaths averted or postponed: 1474 (UI = 220–4995)	–
Hendriksen et al. (2015) [22]	Mathematical/Statistical	Not modelled	Selected foods contributing to high intakes of NaCl	↓50% in NaCl content on average	Mandatory	0.9 Na g/day (37%)	–	–	–
Nghiem et al. (2015) [46]	Markov	Cohort life-time	All processed foods	↓25% in NaCl	Mandatory	0.525 Na g/day (15%)	–	–	4783 QALYs gained/100000 pop (UI = 3804–7174)
			Breads, processed meats and sauces	↓25% in NaCl	Mandatory	0.296 Na g/day (9%)	–	–	2683 QALYs gained/100000 pop (UI = 2161–3256)
Wilcox et al. (2015) [34]	Epidemiological	10y	Not modelled	Not modelled	Mandatory	0.005 Na g/day (10%) (UI = 0.003–0.021)	1.15 mmHg (UI = 0.57–4.58)	CHD Deaths averted: 497 (UI = 130–3032)	–
								LYG: 11192 (UI = 5679–41,039)	–
Collins et al. (2014) [36]	Epidemiological	10y	Not modelled	↓15% in NaCl content overall	Voluntary	1.21 Na g/day (UI = 0.32–1.94)	–	LYG: 14593 (UI = 9000–21,049)	–
				↓20% in NaCl content overall	Mandatory	1.62 Na g/day (UI = 0.65–3.11)	–	LYG: 19365 (UI = 11,967–27,887)	–
Hendriksen et al. (2014) [47]	Markov	20y (clinical outcomes); cohort life-time (DALYs)	All processed foods	↓50% in NaCl content on average	Mandatory	2.3 Na g/day (28%)	1.5 mmHg (1.2%)	4.4% AMI (UI = 3.1–5.6%)	0.5% DALYs averted in the population (UI = 0.37–0.68%)
								CHF: 1.8% (UI = 1.3–2.3%)	
								Strokes: 6% (UI = 4.1–7.8%)	
								Increase in life expectancy: 0.7% (UI = 0.5–0.9%)	
Mason et al. (2014) [53]	Epidemiological	10y	Not modelled	Not modelled	Mandatory	10% daily Na intake (UI = 5–40%)	–	Tunisia: LYG 2272 (UI = 1151–3361)	–
								Syria: LYG 11192 (UI = 5679–41,039)	
								Palestine: LYG 945 (UI = 479–3479)	
								Turkey: LYG 135221 (UI = 68,816–487,712)	
Konfino et al. (2013) [37]	Markov	10y	All processed foods	↓8% in NaCl intake (stepped reduction by 4% for the first 2y)	Mandatory (80% of sodium from processed foods)	0.353 Na g/day	1.00–2.00 mmHg	Total Deaths: 0.61%	–
								Fatal CHD: 0.98%	
								AMI: 1.48%	
								Strokes: 0.99%	
				↓40% in NaCl intake (4% per year for 10y)	Mandatory (80% of sodium from processed foods)	1.763 Na g/day	5.00–9.00 mmHg	Total Deaths: 1.77%	–
								Fatal CHD: 2.63%	
								AMI: 4.27%	
								Strokes: 2.79%	
Bertram et al. (2012) [38]	Epidemiological	1y	Bread, margarine, gravy, soups	↓54% in NaCl content on average	Mandatory	0.85 Na g/day	–	Strokes: 8%	–
								CHD: 6.5%	
								Hypertensive heart disease: 11%	
Cobiac et al. (2012) [48]	Markov	Cohort life-time	Bread, margarine, breakfast cereals	Based on Heart Foundation Tick Programme: ↓26% in NaCl content in bread; 11% in margarine and 61% in breakfast cereals	Mandatory	0.009 Na g/day	–	–	1451 DALYs averted/100000 pop (UI = 1088–1813)
Combris et al. (2011) [8]	Mathematical/Statistical	Not modelled	Breakfast cereals	Mild to strong reformulation based on food nutrient distribution	Mandatory	0.001–0.013 Na g/day (1.4–13.5%)	–	–	–
			Biscuits/ pastries			0.0003–0.002 Na g/day (1.70–10.81%)	–	–	–
			Bread-based products			0.0023–0.013 Na g/day (1.60–8.8%)	–	–	–
Cobiac et al. (2010) [49]	Epidemiological	Cohort life-time	Bread, margarine, breakfast cereals	Based on Heart Foundation Tick Programme: ↓26% in NaCl content in bread; 11% in margarine and 61% in breakfast cereals	Voluntary	0.009 Na g/day	–	–	5300 DALYs averted (UI = 2600–9200)
					Mandatory extension of actual program to all products	–	–	–	110,000 DALYs averted (UI = 53,000–180,000)
Smith-Spangler et al. (2010) [50]	Markov	Cohort life-time	Not modelled	Not modelled	Voluntary	9.5% daily Na intake (UI = 5–40%)	1.25 mmHg	Strokes averted: 513885	2,060,790 DALYs averted
								AMI averted: 480538	
Roodenburg et al. (2009) [27]	Mathematical/Statistical	Not modelled	All processed foods	Reformulation set to meet Choices Programme criteria	Mandatory	23% daily Na intake (10% adjusting for energy compensation)	–	–	–
Rubinstein et al. (2009) [51]	Markov	Cohort life-time	Bread	↓ to 1 g of NaCl per 100 g of bread	Voluntary	–	1.33 mmHg	–	18.7 DALYs averted/100000 pop
Murray et al. (2003) [52]	Markov	Cohort life-time	Not modelled	Not modelled	Mandatory	Assumed 30% Na Intake	AmrB: 3.11% on average	–	600,000 DALYs averted
							EurA: 3.49% on average	–	1,300,000 DALYs averted
							SearD: 3.49% on average	–	1,000,000 DALYs averted
					Voluntary	Assumed 15% Na Intake	AmrB: 1.56% on average	–	300,000 DALYs averted in the population
							EurA: 1.74% on average	–	700,000 DALYs averted in the population
							SearD: 1.75% on average	–	500,000 DALYs averted in the population

(Abbreviations: *AMI* – Acute Myocardial Infarction, *AmrB* – Region of the Americas group B, *CHD* – Coronary Heart Disease, *CHF* – Coronary Heart Failure, *CVD* – Cardiovascular diseases, *DALY* – Disability Adjusted Life Years, *EurA* – European Region group A, *FDII* – Food and Drink Industry Ireland, *F&V* – fruit and vegetables, *K* – potassium, *LYG* – Life Years Gained, *Mg* – magnesium, *Na* – sodium, *NaCl* – Sodium Chloride, *NL* – Netherlands, *NSRI* – National Salt Reduction Initiative, *QALY* – Quality Adjusted Life Year, *SearD* – Southeast Asian Region group D, *UI* – Uncertainty Interval, *UK* – United Kingdom, *US* – United States of America)

**Table 2:** – Interventions targeting sugar consumption

Author (year)	Study Characteristics					Study Outcomes		
	Model type	Time Horizon	Target foods	Type of intervention(s)	Voluntary/mandatory	Reduction in individual intake	Reduction in weight	Reduction in the incidence of clinical outcomes
Briggs et al. (2017) [39]	Epidemiological	1y	High and mid-sugar drinks	↓30% in sugar for high-sugar drinks; 15% for mid-sugar drinks	Mandatory	5.38 sugar g/day (27.5%, UI = 4.19–5.76) - i.e. about 21 Kcal/day	–	T2D incidence: 31.1 per 100,000 persons (UI = 11–53)
								Obesity prevalence: 0.9% (UI = 0.3–19%)
				↓5% in sugar content for both high and mid-sugar drinks	Mandatory	0.98 sugar g/day (5%, UI = 0.92–1.05) - i.e. about 3.92 Kcal/day	–	T2D incidence: 5.8 per 100,000 persons (UI = 2–10)
								Obesity prevalence: 0.2% (UI = 0.09–4%)
Yeung et al. (2017) [28]	Mathematical/statistical	Not modelled	Selected foods with at least 5 g of added sugars/100 g	↓10% in added sugar	Mandatory	10.75 Kcal/day (SE = 36, 0.52%) in 2-16y	–	–
						4.62 added sugar g/day (SE = 2.9, 7.69%) in 2-16y		
				↓15% in added sugar	Mandatory	16.25 Kcal/day (SE = 55, 0.79%) in 2-16y	–	–
						6.97 added sugar g/day (SE = 4.47, 11.59%) in 2-16y		
				↓25% in added sugar	Mandatory	27.24 Kcal/day (SE = 92, 1.34%) in 2-16y	–	–
						11.73 added sugar g/day (SE = 7.5, 19.5%) in 2-16y		
Food and Drink Industry Ireland (2016) [26]	Mathematical/statistical	Not modelled	10 Food macrocategories	Reformulation based on actual FDII voluntary programme	Mandatory extension of existing programme	1.02 sugar g/day (1.12%) in adults	–	–
					Voluntary	0.27 sugar g/day (0.30%) in adults	–	–
Leroy et al. (2016) [32]	Epidemiological	1y	F&V, bread, meat, fish, sandwiches, sauces	Strong reformulation based on the Choices Programme criteria	Mandatory	14.4% daily sugar intake	–	Fatal CVD/Strokes deaths averted: 421
								Cancer deaths averted: 324
				Mild reformulation based on the Choices Programme criteria	Mandatory	4.6% daily sugar intake	–	CVD/Strokes and Cancer deaths averted: 2408 (3.7%) - due to total reductions in Na, SFA and sugar consumption combined
Ma et al. (2016) [40]	Mathematical/statistical	5y	Sugar sweetened beverages (with juices)	↓40% in added sugar content (9.7% per year over 5 years)	Mandatory	38.4 Kcal/day (UI = 36.3–40.7)	1.2 kg (UI = 1.12–1.28)	BMI reduction: 0.42 kg/m^2^ (1.5%)
								Overweight prevalence: 1%
								Obesity prevalence: 2.1%
								T2D incidence: 274000–309,000 cases averted
			Sugar sweetened beverages (without juices)			31.0 Kcal/day (UI = 28.6–33.7)	0.96 kg (UI = 0.88–1.04)	BMI reduction: 0.34 kg/m^2^(1.2%)
								Overweight prevalence: 0.7%
								Obesity prevalence: 1.7%
								T2D incidence 221,000–250,000 cases averted
Masset et al. (2016) [25]	Mathematical/statistical	Not modelled	Pizza	Reformulation to meet Nestlè Nutrient Profiling targets	Mandatory	0.1 sugar g/day (0.1%)	–	–
Combris et al. (2011) [8]	Mathematical/statistical	Not modelled	Breakfast cereals	Mild to strong reformulation based on food nutrient distribution	Mandatory	0.125–0.278 sugar g/day (1.80–4%)	–	–
			Biscuits/ pastries			0.006–0.068 sugar g/day (0.30–3.5%)	–	–
			Bread-based products			0.058–0.288 sugar g/day (2.80–13.9%)	–	–
Hendriksen et al. (2011) [41]	Mathematical/statistical	Not modelled	Carbonated soft drinks	100% substitution of sugar with intense sweeteners	Mandatory	80.5 Kcal/day	3.55 kg	Obesity prevalence: 4%
								BMI reduction: 1.5 kg/m^2^
Roodenburg et al. (2009) [27]	Mathematical/statistical	Not modelled	All processed foods	Reformulation set to meet Choices Programme criteria	Mandatory	37% daily sugar intake (29% adjusting for energy compensation)	–	–
Husøy et al. (2008) [24]	Mathematical/statistical	Not modelled	Carbonated soft drinks	100% substitution of sugar with intense sweeteners	Mandatory	36.5% energy intake	–	–

Abbreviations: BMI – Body Mass Index; CVD – Cardiovascular diseases; FDII – Food and Drink Industry Ireland; F&V – fruit and vegetables; SE – Standard Error; T2D – Type 2 diabetes; UI – Uncertainty Interval

**Table 3:** – Interventions targeting fat consumption

Author (year)	Study Characteristics					Study Outcomes		
	Model type	Time horizon	Target foods	Type of intervention(s)	Voluntary/mandatory	Reduction in individual intake	Reduction/increase in the incidence of clinical outcomes	Results on QOL measures
Food and Drink Industry Ireland (2016) [26]	Mathematical/statistical	Not modelled	10 Food macrocategories	Reformulation based on actual FDII voluntary programme	Mandatory extension of existing programme	1.67 SFA g/day (5.5%) in adults	–	–
					Voluntary	0.47 SFA g/day(1.50%) in adults	–	–
Leroy et al. (2016) [32]	Epidemiological	1y	F&V, bread, meat, fish, sandwiches, sauces	Strong reformulation based on the Choices Programme criteria	Mandatory	14.8% daily SFA intake	Fatal CVD/Strokes deaths averted: 1339	–
							Cancer deaths averted: 558	–
				Mild reformulation based on the Choices Programme criteria	Mandatory	11.7% daily SFA intake	CVD/Strokes and Cancer: 2408 deaths averted (3.7%) - due to total reductions in Na, SFA and sugar consumption combined	–
Masset et al. (2016) [25]	Mathematical/statistical	Not modelled	Pizza	Reformulation to meet Nestlè Nutrient Profiling targets	Mandatory	0.3 SFA g/day (1.1%)	–	–
Pearson-Stuttard et al. (2016) [33]	Epidemiological	10y	All processed food	↓100% (Total ban) on industrial TFA	Mandatory	↓100% industrial TFA ➔ approx. 0.4% of daily energy intake from ruminant TFA	CHD deaths averted or postponed: 1700 (UI = 1619–1825)	–
							LYG: 15000 (UI: 13952–16,934)	–
Allen et al. (2015) [44]	Epidemiological	6y	All processed foods	↓100% (Total ban) on industrial TFA	Mandatory	↓100% industrial TFA ➔ approx. 0.4% of daily energy intake from ruminant TFA	CHD deaths averted or postponed: 7200 (UI = 3200–12,500; 2.6%)	7900 QALYs gained (UI = 3000–13,900)
Combris et al. (2011) [8]	Mathematical/statistical	Not modelled	Breakfast cereals	Mild to strong reformulation based on food nutrient distribution	Mandatory	0.032–0.172 fat g/day (1.40–7.5%)	–	–
			Biscuits/ pastries			0.0162–0.061 fat g/day (1.40–5.30%)	–	–
			Bread-based products			0.009–0.229 fat g/day (0.40–9.90%)	–	–
Temme et al. (2011) [11]	Mathematical/statistical	Not modelled	Potato-products, bread, pastry, cakes and biscuits (excluding foods made with butter); (meat) snacks and salads, fats and margarines	↓ 300% TFA in potato products for frying; 33% in bread; 75% in pastry, cakes and biscuits; 67% for meat snacks and salads	Mandatory	0.4 TFA g/day (21.1%, UI = 0.35–0.45)	–	–
Roodenburg et al. (2009) [27]	Mathematical/statistical	Not modelled	All packaged foods	Reformulation set to meet Choices Programme criteria	Mandatory	40% SFA daily intake (32% adjusting for energy compensation)	–	–
						63% TFA daily intake (58% adjusting for energy compensation)	–	–

Abbreviations: *CHD* – Coronary Heart Disease, *CVD* – Cardiovascular diseases, *FDII* – Food and Drink Industry Ireland, *F&V* – fruit and vegetables, *LYG* – Life Years Gained, *Na* – sodium, *QALY* – Quality Adjusted Life Year, *SFA* – Saturated Fatty Acids, *TFA* – Trans Fatty Acids: UI – Uncertainty Interval

Target foods in the included studies were all processed foods (*n* = 9) [27, 31, 33, 37, 38, 41, 45, 51, 52], selected groups of nutrient-dense foods (*n* = 8) [11, 26, 34–36, 43, 44, 50], or single food products (*n* = 8) [23, 24, 42, 46–49]. Four studies provided estimates for more than one item [8, 28–30], whereas in 4 studies target foods were not specified since reformulation was modelled directly through its assumed effect on intake [22, 25, 32, 39]. Ten studies simply estimated changes in intake following reformulation (30.3%) [8, 11, 26, 33, 42, 43, 45, 49, 50, 53], whereas other studies estimated effects on health outcomes (*n* = 12, 36.4%) [25, 31, 35, 37, 39–41, 44, 46–48, 51] or health related quality of life measures (*n* = 11, 33.3%) [22–24, 28–30, 32, 34, 36, 38, 52].

Ten studies used epidemiological models linking changes in nutrients intake to changes in disease incidence and prevalence at an established time [25, 34, 35, 39–41, 44, 47, 52]. These studies include comparative risk assessment models (e.g. WHO Comparative Risk Assessment, or PRIME) [35, 47], potential impact fraction models [34] and other validated models, including DIETRON [44], and IMPACT models [25, 39–41, 51, 52]. Ten studies used cohort Markov models [22, 23, 28–30, 32, 36–38], or individual level micro-simulations [31]. Finally, 13 records, accounting for almost 40% of the included studies, used mathematical-statistical models to estimate the potential change in intake by linking cross-sectional, individual-level consumption data to the nutrient-density of foods before and after reformulation [8, 11, 24, 26, 33, 42, 43, 45, 46, 48–50, 53]. In the majority of this last group, time was not explicitly considered, since the analysis was limited to a re-assessment of past individual intakes after reformulating all or a specific set of products. In the remaining studies, the time horizon of the analysis was highly variable: 5 studies considered a time ranging from 1 to 6 years [35, 44, 46, 47, 52], 8 studies between 10 and 20 years [25, 31, 37–41, 51] and 9 studies considered the average lifetime of the modelled population [22–24, 28–30, 32, 34, 36].

Strikingly, 30% of the studies were not clear about what informed the amounts of nutrient reformulated, and whether considerations were made on technical feasibility, or other aspects such as shelf life and palatability.

Most of the studies explored the impact of likewise mandatory policies, i.e. they assumed that all target foods would be reformulated, while only 5 studies performed scenario analysis modelling both voluntary and mandatory reformulation. This was usually done by assuming smaller proportions of reformulated products in the voluntary scenario [34, 41, 43], or by assuming longer implementation times and/or more uncertain effects on intake [22, 29].

Only 2 studies performed scenario analysis to explore the interplay between reformulation policies and consumers’ reactions. Roodenburg et al. tested the results of the model against the possibility that consumers maintained the same caloric intake by eating more food [45], whereas Choi et al. considered whether results were robust to the possibility that consumers might add discretionary salt, or switch to more salty alternatives after introducing low-salt reformulations [31].

### Interventions aiming to reduce sodium intake

Reported percentages of sodium reductions in the models varied between 11 and 63% across different products, focusing particularly on bread, sauces and processed meats (Table 1).

**Fig. 3:**
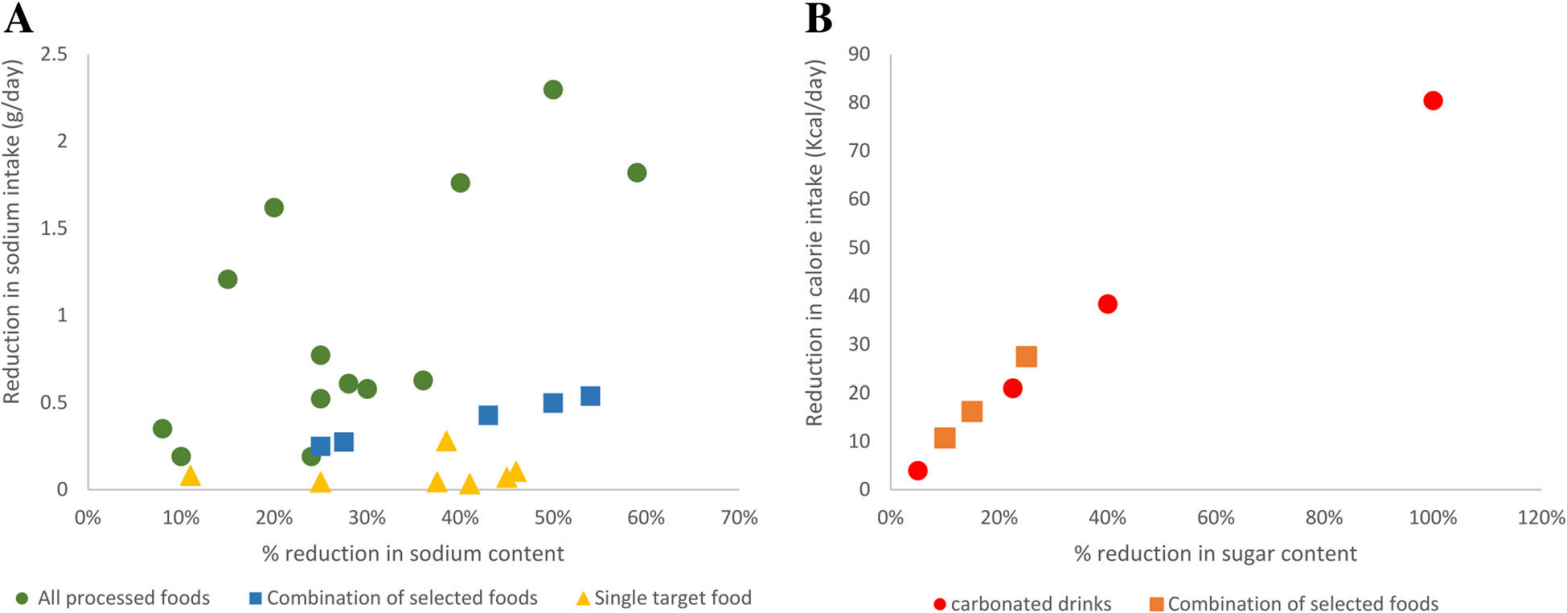
Relationship between amounts reformulated and individual intakes of sodium and sugar. Scatter plot of studies reporting the effects of % reductions in the nutrient content of food on sodium intake (g/day, panel **a**), and energy intake for sugar reformulations (Kcal/day, panel **b**)

Studies reporting absolute reductions in sodium intake showed effects ranging from 0.009 to 1.82 g/day per person, mainly depending on the amount of nutrients reformulated, the spectrum of targeted foods and scenario studied [30, 36]. Overall, a certain degree of consistency was found across studies, with higher percentages of sodium reformulated leading to higher reductions in sodium intake, which in addition tend to be more marked for interventions addressing all processed foods compared to narrower sets of products (Fig. 3, panel A).

Studies estimating the effects on health outcomes showed a percentage reduction in CVD-related mortality in a range between 0.6 and 1.7% [31, 37], stroke incidence between 0.5 and 8% [24, 31, 35, 37, 38], and Acute Myocardial infarction (AMI) incidence between 0.3 and 4.4% [24, 31, 37, 38].

Most studies reported the number of deaths averted, life year gains and quality of life measures (QALYs or DALYs) in absolute values. This limited between-study comparisons, since results were sensitive to the time horizon of the analysis, the size of the modelled population, and baseline risk factors, which varied across studies. In the studies for which it was possible to calculate gains per 100,000 population, reductions in the sodium content of foods resulted in an increase between 265 QALYs and 12,783 QALYs [28–30], or in a reduction between 6.35 and 1452 DALYs [23, 24, 36]. Although based on less studies, results seem to be consistent across models, showing a positive association between sodium reductions and QALYs (DALYs) gained (lost).

### Interventions to reduce intake of sugar

Reformulation to reduce sugar intake targeted Sugar Sweetened Beverages (SSBs) alone [46–49], or other sets of foods ranging from pizza to all processed foods [8, 42–45, 50] (Tables 2).

Similarly to sodium strategies, although based on fewer studies, reductions in energy intake (Kcal per day) for SSBs are consistent across studies and proportional to the amount of sugar reformulated (Fig. 3, panel B). For example, Briggs et al. estimate that a reduction in the sugar content of SSBs by 5 and 23% would reduce calorie intake by about 4 Kcal and 21 Kcal per day [47]. Likewise, when cutting 10 to 25% of sugar content in selected sugar-dense foods, Yeung et al. estimated a reduction in energy intake from 11 to 27 Kcal per day [50].

Few studies modelled the impact of reformulating sugar on health outcomes. Prevalence of obesity is estimated to be reduced in a range between 0.2 and 4% [46–48], whereas one study estimated a reduction in type 2 diabetes between 5.8 and 31.1 incident cases per 100,000 persons [47]. A further study estimated that a broader reformulation policy, designed to comply with the Dutch Choices Program for selected processed foods, would yield a reduction in mortality caused by chronic disease between 3.7 and 5.5% [44]. Lastly, no studies were found that estimated the impact of reformulation on health related quality of life measures.

### Interventions to reduce intake of fat

Studies addressing reformulation of SFA or TFA are limited. Temme et al., estimated that a broad intervention cutting TFA on a set of products would lower trans-fat consumption by 0.4 g/day (21.1%, UI = 0.35, 0.45) (Table 3) [11]. In addition, two studies estimated that banning all industrial TFA from processed foods would avert 1700 to 7200 deaths and generate a gain of 7900 QALYs [51, 52].

Other interventions modelling broader multi-nutrient reformulations for different target products found percentage reductions in SFA consumption to be in a range between 1.1 and 40% [43–45] (Table 2). One single study estimated the effect of fat reformulation on mortality, showing that if all producers in the food industry complied with the International Choices Programme [27], SFA consumption would reduce by 15%, which in turn would reduce deaths by 3% (1339 deaths from cardiovascular diseases and 558 deaths from cancer) [44].

### Quality of studies

**Fig. 4:**
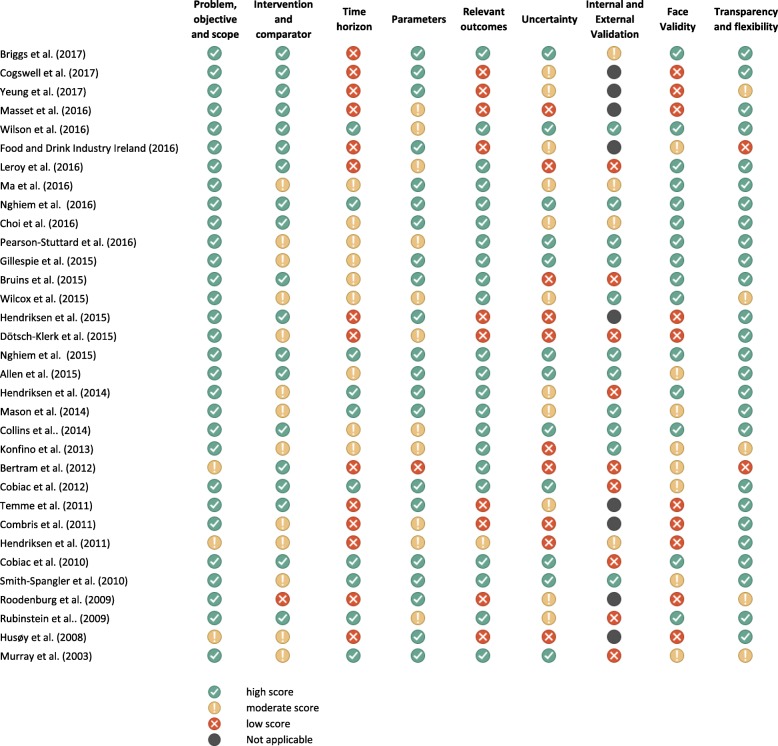
Quality assessment of the included studies

Most of the studies (64%) scored high in at least 50% of the considered assessment elements, whereas 9% of studies had mainly moderate scores and 27% received low scores. Study quality was generally satisfactory in the criteria relating to the description of the problem, the research objective and the scope (91% of the included studies); the transparency and provision of technical documentation (79%); the type of outcomes included (67%). However, studies generally scored poorly when assessing the adequacy of the time horizon (42% low and 27% moderate scores), the management and reporting of uncertainty in the model parameters (30% low, 33% moderate) and the internal and external validation (38% low, 17% moderate). Lastly, only 45% of the studies scored high in face validity that is their results were considered credible and realistic (Fig. 4). Particularly, studies were considered to have high face validity if they duly and credibly took into account all aspects of the decision problem, including the technical feasibility of the reformulation scenarios, and all the causal steps linking the intervention to the outcomes of interests (e.g. the reactions of the consumers and manufacturers to the intervention).

## Discussion

The present systematic review collected and synthetized the results of simulation models estimating the effect of reformulation policies to improve population diets. Most of the studies focused on sodium, which is not unexpected since sodium reduction strategies have been a policy priority for much longer compared to those for sugar and fats. All models predicted positive outcomes; however, between-study comparability was limited, especially for health outcomes and health related quality of life measures. In addition, no studies estimated long-term consequences on QALYs or DALYs for sugar, TFA, SFA, or broader multi-nutrient reformulations. More research is needed to evaluate the potential impact of reformulation strategies in achieving national and international consumption targets, and ultimately improving public health.

In addition, more evidence is required on the relative effectiveness and cost-effectiveness of reformulation compared to alternative public health nutrition interventions (e.g. food taxes, public media campaigns or food labelling).

The effectiveness of reformulation strategies on public health is the result of a complex causal chain that, besides biological factors linking intake to health outcomes, it also includes technical/industrial aspects, marketplace dynamics and consumer reactions. The incorporation of these aspects relates to the face validity of the model, that is defined as the extent to which a model plausibly represents the diseases, settings, populations, interventions, and outcomes it is intended to analyze [21].

For 30% of the included studies, strategies were not clearly informed by aspects related to the technical feasibility of reformulation, including the essential requirements to preserve shelf-life, volume and palatability. However, since the amounts of nutrients substituted in the scenarios directly affect the estimated impact on intake and health, the nature and the intensity of reformulation strategies should always be defined based on real observed reductions from on-going initiatives, or informed by reliable evidence or expert opinions.

In addition, reformulation can alter the sensory attributes of food products and influence consumer liking [54, 55]. This in turn may trigger unattended behaviors including the consumption of more public health sensitive nutrients, or simply more calories. Nonetheless, only two of the included studies explicitly modelled how consumer reactions might affect the policy effects. Choi et al. estimated that the beneficial effects of a mandatory reformulation policy would remain even if the use of discretionary salt increased by 15% [31]. However, such threshold percentage may be lower for less pervasive or voluntary reformulation policies. Roodenburg et al. tested the results of the model against the possibility that consumers maintained the same caloric intake by eating more food [45]. They found that, after this adjustment, differences in intakes for energy, SFA, TFA, sodium and sugar would be smaller. In addition, while there is evidence that consumers gradually adapt to changes in the salt content of foods [56, 57] and that small salt reductions in certain products cannot be detected and do not affect acceptability or consumption [34, 58, 59], this is more controversial for fat [60] and sugar [61].

With respect to marketplace dynamics, unsurprisingly, in the few studies where both mandatory and voluntary interventions were modelled, mandatory scenarios were always found to be more effective. Nevertheless, such differences may partly reflect various assumptions around intervention phase-in periods [29], or the considered rate of adherence from the industry. For example, when allowing for the possibility that consumers switched to non-reformulated products, Choi et al. estimate that significant reductions in CVD mortality would occur only if more than 65% of products in the market met the reduction criteria [31].

Anticipating how food manufacturers and consumers will react to a reformulation initiative are core components of models, so that lack of consideration for these aspects may undermine the credibility of the model results. Since there may be little data on these parameters at the time of the assessment, studies should clearly report and justify the underlying assumptions made in their models, and conduct extensive sensitivity analysis to test the robustness of results at different levels of industry uptake and consumers reactions. In addition, in some cases, real world sources can be used to model more realistic scenarios. A study by Temme et al. assessing the impact on intake of foods reporting a health logo estimated the expected consumption rate of healthy and unhealthy products, by looking at real market shares of products with a healthy logo over the total purchases in each food category [62].

The time horizon considered in reformulation models should be long enough to account for all relevant consequences of the interventions, including the long-term health effects of improved dietary patterns. In addition, many aspects of the reformulation models can vary over time including the industry uptake of reformulated products, consumption habits and preferences, and secular epidemiological trends in non-communicable diseases. In this review, 40% of the studies did not explicitly model the effects of the interventions over time and focused on simulating how cross-sectional nutrient intake data would change if foods were reformulated. Although consistent with their declared research questions, these studies do not estimate how the modelled interventions will dynamically affect food purchases, intake, and ultimately health outcomes. The dynamic nature of public health interventions and the presence of complex, interdependent factors have already been pointed out in the literature [63–65] calling for more methodological developments, such as the use of system dynamics modelling to incorporate time dependencies [66].

Besides face validity, other standard steps of validation apply to reformulation models [21]. Internal validity should be verified by demonstrating that the model behaves as intended and has been implemented correctly. In addition, whenever possible, the simulated model outcomes should be confronted with real-world event data. External validation involves that the entire model or any of its components are verified by confronting the forecasted estimates with actual event data. For example, epidemiological studies or trial data could be used to verify the correctness of the simulated incidence of non-communicable diseases in the absence of reformulation. In addition, models should prove to be able to correctly predict future outcomes for the specific setting, population and intervention of interest. Therefore, when possible, the results of the model should be compared ex-post with the real-world outcomes, should the modelled intervention be implemented as planned. However, assessing the predictive ability of a model for medium and long term effects such as health and quality of life is challenging, as it would require longitudinal data with a rigorous counterfactual scenario [67]. While this type of evidence is often missing, partial validation could be achieved by assessing the goodness of models in predicting intermediary effects such as intakes or surrogate health outcomes (e.g. hypertension).

External and predictive validation are critical for simulation models as their main purpose is primarily to help decision makers anticipate what will occur after introducing a certain policy [68]. Therefore, models should be transparent on how validation is verified. However, only one third of the studies used previously validated models or reported methods used to validate their own models. A more careful consideration of validation would improve study quality and increase trust and confidence in models to inform policy-making.

This review reported the potential effects of reformulation policies on intake and health and proposed an ad-hoc tool to assess the quality of modelling studies on reformulation. .

Nonetheless, a number of limitations are outlined: studies were very different in the way policy effects were modelled and reported so that the provided ranges of effectiveness should be considered with caution; this heterogeneity is a reflection of the lack of guidelines and standardized methods for the evaluation of public health interventions in general. Methods for the evaluation of healthcare interventions have indeed existed for several years [63, 68–72], but these have mainly been applied to more narrowly-defined ‘clinical’ interventions, such as drugs, devices and medical procedures [64]. In contrast, approaches in the field of nutrition and public health are not framed by common objectives, shared methods and/or a strong regulatory environment. Therefore, the establishment of an agreed framework specifying best modelling practices is needed to improve the methodological and reporting quality, as well as the comparability of studies evaluating public health interventions in general and nutrition interventions in particular.

Finally, the number of available studies and the described heterogeneity did not allow to perform subgroup analyses to verify whether specific model features or study quality impact their results. Particularly, in this study, we did not assess existing sources of variations in the effectiveness of reformulation and other nutrition interventions between and within countries, including biological, cultural, socio-economic and institutional factors. Future work may explore the contribution of these aspects on each link of the causal chain from intervention to public health outcomes. Explaining cross-country variations may help to understand which factors are favoring or hindering the effectiveness of nutrition policies at the global level, and their role in reducing the burden of non-communicable diseases. Explaining within-country variations may contribute to incorporate equity considerations in public decision making about nutrition policies at the national level.

## Conclusions

Reformulation policies have the potential to improve diets and population health. Evidence is stronger for sodium interventions, but far less conclusive for sugar and fat reformulations. Mathematical models are valuable tools to predict policy effects, although comparability is often limited by different study designs, assumptions and reporting quality. More homogeneous designs and assumptions, combined with the validation of model results and extensive scenario analysis to evaluate the relevance of specific policy features, would improve model credibility and provide policy-makers with useful insights to design evidence-based nutrition policies.

## Additional files

Additional file 1: PRISMA checklist. (DOCX 21 kb)

Additional file 2: Search strategy on Medline (via Web of Science). (DOCX 16 kb)

Additional file 3: Quality assessment tool. (DOCX 22 kb)

Additional file 4: Additional study data. (DOC 104 kb)

## Abbreviations

AMI: Acute myocardial infarction
BMI: Body mass index
CHF: Congestive heart failure
CVD: Cardiovascular diseases
DALY: Disability adjusted life years
LYG: Life-years gained
QALY: Quality adjusted life years
SFA: Saturated fatty acids
SSB: sugar sweetened beverage
T2D: Type 2 diabetes
TFA: Trans-fatty acids
UI: Uncertainty interval
