# Correction to: The impact of food reformulation on nutrient intakes and health, a systematic review of modelling studies

**DOI:** 10.1186/s40795-019-0269-8

**Published:** 2019-02-04

**Authors:** Carlo Federici, Patrick Detzel, Francesco Petracca, Livia Dainelli, Giovanni Fattore

**Affiliations:** 1 CeRGAS (Centre for Research on Health and Social Care Management), SDA Bocconi School of Management, Milan, Italy; 2 Nestlé Research Center, Nestec SA, Lausanne, Switzerland; 3 Department of Policy Analysis and Public Management, Bocconi University, Milan, Italy

**Correction to: BMC Nutr (2019) 5:2**

**https://doi.org/10.1186/s40795-018-0263-6**

Following publication of the original article [1], the authors reported an error in Table 1. The rows and columns do not align correctly. The correct table can be found below.

**Table 1:** Interventions targeting sodium consumption

Author (year)	Study Characteristics					Study Outcomes			
	Model type	Time horizon	Target foods	Type of intervention(s)	Voluntary or mandatory	Reduction in intake	Reduction in blood pressure (BP)	Life years gained and reduction in the incidence of health outcomes	Results on QOL measures
Cogswell et al. (2017) [29]	Mathematical/Statistical	Not modelled	All processed food	↓28% in Na content (Health Canada Benchmarks)	Mandatory	0.61 Na g/day (22%, UI = 0.59–0.63)	–	–	–
Choi et al. (2016) [30]	Micro-simulation	10y	All processed foods	Product reformulated to meet product-specific NSRI criteria extended to all food producers in the US	Mandatory	0.365 (SE = 0.9) Na g/day (10.9%)	–	Hypertension: 0.97%	–
								All AMI: 2.59%	
								All strokes: 2.67%	
								Fatal AMI: 0.36%	
								Fatal Strokes: 0.23%	
Food and Drink Industry Ireland (2016) [26]	Mathematical/Statistical	Not modelled	10 Food macrocategories	Reformulation based on actual FDII voluntary programme	Mandatory extension of existing programme	0.57 Na g/day (17.8%) in adults	–	–	–
					Voluntary	0.06 Na g/day (2.3%) in adults	–	–	–
Leroy et al. (2016) [32]	Epidemiological	1y	F&V, bread, meat, fish, sandwiches, sauces	Strong reformulation based on the Choices Programme criteria	Mandatory	12.7% daily Na intake	–	Fatal CVD/Strokes deaths averted: 422	–
								Cance deaths averted: 187	
				Mild reformulation based on the Choices Programme criteria	Mandatory	9.3% daily Na intake	–	CVD/Strokes and Cancer deaths averted: 2408 (3.7%) - due to total reductions in Na, SFA and sugar consumption combined	–
Masset et al. (2016) [25]	Mathematical/Statistical	Not modelled	Pizza	Reformulation to meet Nestlè Nutrient Profiling targets	Mandatory	0.14 Na g/day (4%)	–	–	–
Nghiem et al. (2016) [42]	Markov	Cohort life-time	All processed foods	59% substitution of NaCl with other salts (K, Mg)	Mandatory	1.82 Na g/day (51.5%)	–	–	12,783 QALYs gained/100000 pop. (UI = 10,348–15,609)
				25% substitution of NaCl with other salts (K, Mg)	Mandatory	0.77 Na g/day (21.8%)	–	–	5261 QALYs gained/100000 pop. (UI = 4230–6391)
			Bread	↓38,5% in NaCl content	Mandatory	0.28 Na g/day (7.9%)	–	–	1891 QALYs gained/100000 pop. (UI = 1509–2296)
				↓11,1% in NaCl content	Mandatory	0.08 Na g/day (2.3%)	–	–	678 QALYs gained/100000 pop. (UI = 548–822)
Wilson et al. (2016) [43]	Markov	Cohort life-time	All processed foods (bread, processed meats, sauces, snack food, bakery, cheese)	↓36% in NaCl content across product types	Mandatory	0.628 Na g/day	–	–	5304 QALYs gained/100000 pop. (UI = 4270–6478)
					Voluntary	Same efficacy with higher uncertainty	–	–	5000 QALYs gained/100000 pop. (UI = 3709–6391)
			Bread	↓12–37% in NaCl content across bread types	Mandatory	0.043 Na g/day	–	–	387 QALYs gained/100000 pop. (UI = 309–470)
					Voluntary	Same efficacy with higher uncertainty	–	–	365 QALYs gained/100000 pop. (UI = 270–461)
			Processed meats	↓35–55% in NaCl content overall	Mandatory	0.069 Na g/day	–	–	583 QALYs gained/100000 pop. (UI = 470–704)
					Voluntary	Same efficacy with higher uncertainty	–	–	552 QALYs gained/100000 pop. (UI = 417–696)
			Sauces	↓30–63% in NaCl content across sauces types	Mandatory	0.104 Na g/day	–	–	870 QALYs gained/100000 pop. (UI = 700–1057)
					Voluntary	Same efficacy with higher uncertainty	–	–	822 QALYs gained/100000 pop. (UI = 626–1039)
			Combination of bread, processed meats and sauces	–	Mandatory	0.217 Na g/day	–	–	1843 QALYs gained/100000 pop. (UI = 1487–2239)
					Voluntary	Same efficacy with higher uncertainty	–	–	1743 QALYs gained/100000 pop. (UI = 1326–2204)
			Snack food	↓34–48% in NaCl content across snacks types	Mandatory	0.032 Na g/day	–	–	265 QALYs gained/100000 pop. (UI = 217–322)
					Voluntary	Same efficacy with higher uncertainty	–	–	252 QALYs gained/100000 pop. (UI = 191–317)
			Bread and bakery products	↓12–37% in NaCl content across bread types; ↓54–63% in NaCl content across other bakery products	Mandatory	0.107 Na g/day	–	–	887 QALYs gained/100000 pop. (UI = 722–1078)
					Voluntary	Same efficacy with higher uncertainty	–	–	843 QALYs gained/100000 pop. (UI = 639–1061)
			Cheese	↓27–42% in NaCl content across cheese types	Mandatory	0.045 Na g/day	–	–	383 QALYs gained/100000 pop. (UI = 309–461)
					Voluntary	Same efficacy with higher uncertainty	–	–	361 QALYs gained/100000 pop. (UI = 274–457)
Bruins et al. (2015) [45]	Mathematical/Statistical	Cohort life-time	Soups	↓25% in Na content	Mandatory	0.05 Na g/day	0.11 mmHg	Strokes: 0.49%	6.45 DALYs averted/100000 pop
								AMI: 0.34%	
								Angina: 0.34%	
								CHF: 0.24%	
Dötsch-Klerk et al. (2015) [23]	Mathematical/Statistical	Not modelled	All processed foods	Products reformulated to meet the 6 g/day NaCl consumption target	Mandatory	US: 1.8 Na g/day (23%)	–	–	–
						UK: 1.8 Na g/day (27%)	–	–	–
						NL: 1.3 Na g/day (19%)	–	–	–
				Products reformulated to meet the 5 g/day NaCl consumption target	Mandatory	US: 2.2 Na g/day (28%)	–	–	–
						UK: 2.1 Na g/day (32%)	–	–	–
						NL: 1.8 Na g/day (26%)	–	–	–
Gillespie et al. (2015) [31]	Epidemiological	10y	All processed foods	↓30% in NaCl	Mandatory	0.58 Na g/day (UI = 0.56–0.60)	0.81 mmHg '(UI = 0.53–1.10)	CHD deaths averted or postponed: 4467 (UI = 2854–6147)	–
				↓10% in NaCl	Mandatory	0.19 Na g/day (UI = 0.18–0.20)	0.27 mmHg (UI = 0.18–0.37)	CHD deaths averted or postponed: 1502 (UI = 953–2068)	–
				↓24% in NaCl	Voluntary (applied to 39% of products)	0.19 Na g/day (UI = 0.03–0.63)	0.27 mmHg (UI = 0.04–0.92)	CHD deaths averted or postponed: 1474 (UI = 220–4995)	–
Hendriksen et al. (2015) [22]	Mathematical/Statistical	Not modelled	Selected foods contributing to high intakes of NaCl	↓50% in NaCl content on average	Mandatory	0.9 Na g/day (37%)	–	–	–
Nghiem et al. (2015) [46]	Markov	Cohort life-time	All processed foods	↓25% in NaCl	Mandatory	0.525 Na g/day (15%)	–	–	4783 QALYs gained/100000 pop (UI = 3804–7174)
			Breads, processed meats and sauces	↓25% in NaCl	Mandatory	0.296 Na g/day (9%)	–	–	2683 QALYs gained/100000 pop (UI = 2161–3256)
Wilcox et al. (2015) [34]	Epidemiological	10y	Not modelled	Not modelled	Mandatory	0.005 Na g/day (10%) (UI = 0.003–0.021)	1.15 mmHg (UI = 0.57–4.58)	CHD Deaths averted: 497 (UI = 130–3032)	–
								LYG: 11192 (UI = 5679–41,039)	–
Collins et al. (2014) [36]	Epidemiological	10y	Not modelled	↓15% in NaCl content overall	Voluntary	1.21 Na g/day (UI = 0.32–1.94)	–	LYG: 14593(UI = 9000–21,049)	–
				↓20% in NaCl content overall	Mandatory	1.62 Na g/day (UI = 0.65–3.11)	–	LYG: 19365(UI = 11,967–27,887)	–
Hendriksen et al. (2014) [47]	Markov	20y (clinical outcomes); cohort life-time (DALYs)	All processed foods	↓50% in NaCl content on average	Mandatory	2.3 Na g/day (28%)	1.5 mmHg (1.2%)	4.4% AMI (UI = 3.1–5.6%)	0.5% DALYs averted in the population (UI = 0.37–0.68%)
								CHF: 1.8% (UI = 1.3–2.3%)	
								Strokes: 6% (UI = 4.1–7.8%)	
								Increase in life expectancy: 0.7% (UI = 0.5–0.9%)	
Mason et al. (2014) [53]	Epidemiological	10y	Not modelled	Not modelled	Mandatory	10% daily Na intake (UI = 5–40%)	–	Tunisia: LYG 2272 (UI = 1151–3361)	–
								Syria: LYG 11192 (UI = 5679–41,039)	
								Palestine: LYG 945 (UI = 479–3479)	
								Turkey: LYG 135221 (UI = 68,816–487,712)	
Konfino et al. (2013) [37]	Markov	10y	All processed foods	↓8% in NaCl intake (stepped reduction by 4% for the first 2y)	Mandatory (80% of sodium from processed foods)	0.353 Na g/day	1.00–2.00 mmHg	Total Deaths: 0.61%	–
								Fatal CHD: 0.98%	
								AMI: 1.48%	
								Strokes: 0.99%	
				↓40% in NaCl intake (4% per year for 10y)	Mandatory (80% of sodium from processed foods)	1.763 Na g/day	5.00–9.00 mmHg	Total Deaths: 1.77%	–
								Fatal CHD: 2.63%	
								AMI: 4.27%	
								Strokes: 2.79%	
Bertram et al. (2012) [38]	Epidemiological	1y	Bread, margarine, gravy, soups	↓54% in NaCl content on average	Mandatory	0.85 Na g/day	–	Strokes: 8%	–
								CHD: 6.5%	
								Hypertensive heart disease: 11%	
Cobiac et al. (2012) [48]	Markov	Cohort life-time	Bread, margarine, breakfast cereals	Based on Heart Foundation Tick Programme: ↓26% in NaCl content in bread; 11% in margarine and 61% in breakfast cereals	Mandatory	0.009 Na g/day	–	–	1451 DALYs averted/100000 pop (UI = 1088–1813)
Combris et al. (2011) [8]	Mathematical/Statistical	Not modelled	Breakfast cereals	Mild to strong reformulation based on food nutrient distribution	Mandatory	0.001–0.013 Na g/day (1.4–13.5%)	–	–	–
			Biscuits/ pastries			0.0003–0.002 Na g/day (1.70–10.81%)	–	–	–
			Bread-based products			0.0023–0.013 Na g/day (1.60–8.8%)	–	–	–
Cobiac et al. (2010) [49]	Epidemiological	Cohort life-time	Bread, margarine, breakfast cereals	Based on Heart Foundation Tick Programme: ↓26% in NaCl content in bread; 11% in margarine and 61% in breakfast cereals	Voluntary	0.009 Na g/day	–	–	5300 DALYs averted (UI = 2600–9200)
					Mandatory extension of actual program to all products	–	–	–	110,000 DALYs averted (UI = 53,000–180,000)
Smith-Spangler et al. (2010) [50]	Markov	Cohort life-time	Not modelled	Not modelled	Voluntary	9.5% daily Na intake (UI = 5–40%)	1.25 mmHg	Strokes averted: 513885	2,060,790 DALYs averted
								AMI averted: 480538	
Roodenburg et al. (2009) [27]	Mathematical/Statistical	Not modelled	All processed foods	Reformulation set to meet Choices Programme criteria	Mandatory	23% daily Na intake (10% adjusting for energy compensation)	–	–	–
Rubinstein et al. (2009) [51]	Markov	Cohort life-time	Bread	↓ to 1 g of NaCl per 100 g of bread	Voluntary	–	1.33 mmHg	–	18.7 DALYs averted/100000 pop
Murray et al. (2003) [52]	Markov	Cohort life-time	Not modelled	Not modelled	Mandatory	Assumed 30% Na Intake	AmrB: 3.11% on average	–	600,000 DALYs averted
							EurA: 3.49% on average	–	1,300,000 DALYs averted
							SearD: 3.49% on average	–	1,000,000 DALYs averted
					Voluntary	Assumed 15% Na Intake	AmrB: 1.56% on average	–	300,000 DALYs averted in the population
							EurA: 1.74% on average	–	700,000 DALYs averted in the population
							SearD: 1.75% on average	–	500,000 DALYs averted in the population

*Abbreviations*: *AMI* Acute Myocardial Infarction, *AmrB* Region of the Americas group B, *CHD* Coronary Heart Disease, *CHF* Coronary Heart Failure, *CVD* Cardiovascular diseases, *DALY* Disability Adjusted Life Years, *EurA* European Region group A, *FDII* Food and Drink Industry Ireland, *F&V* fruit and vegetables, *K* potassium, *LYG* Life Years Gained, *Mg* magnesium, *Na* sodium, *NaCl* Sodium Chloride, *NL* Netherlands, *NSRI* National Salt Reduction Initiative, *QALY* Quality Adjusted Life Year, *SearD* Southeast Asian Region group D, *UI* Uncertainty Interval, *UK* United Kingdom, *US* United States of America

The publishers apologise for this error. The original article [1] has been updated.
