# Supplementary material for: The impact of food reformulation on nutrient intakes and health, a systematic review of modelling studies
Source: BMC Nutr. 2019 Jan 7;5:2. doi: 10.1186/s40795-018-0263-6 (PMC7050744; doi:10.1186/s40795-018-0263-6)
Supplement: Supplementary file 2 — Search strategy on Medline (via Web of Science). (DOCX 16 kb) [file 40795_2018_263_MOESM2_ESM.docx]

## Additional file 2 - Search Strategy used for Medline via Web Of Science

| #11 | #10 |
| --- | --- |
|  | Refined by: **LANGUAGES:** (ENGLISH) |
|  | *DocType=All document types; Language=All languages;* |
| #10 | #9 OR #6 |
|  | *DocType=All document types; Language=All languages;* |
| #9 | #8 AND #7 |
|  | *DocType=All document types; Language=All languages;* |
| #8 | TS=(intake OR consumption OR diet* OR policy OR policies OR intervention*) |
|  | *DocType=All document types; Language=All languages;* |
| #7 | TS=((decreas* OR limit* OR reduce OR reducing OR reduction* OR reformulat* OR formulat* OR redevelop* OR restrict* OR replacement* OR replacing) NEAR/5 (food OR sodium* OR salt* OR sugar* OR fat OR fats OR TFA OR trans NEAR/1 fatty)) |
|  | *DocType=All document types; Language=All languages;* |
| #6 | #5 AND #1 |
|  | *DocType=All document types; Language=All languages;* |
| #5 | #4 OR #3 OR #2 |
|  | *DocType=All document types; Language=All languages;* |
| #4 | MH=(health policy OR nutrition policy) |
|  | *DocType=All document types; Language=All languages;* |
| #3 | MH=(food habits OR food quality OR recommended dietary allowances) |
|  | *DocType=All document types; Language=All languages;* |
| #2 | MH=(Food, Formulated OR Food-Processing Industry OR Food Technology OR Food Industry ) |
|  | *DocType=All document types; Language=All languages;* |
| #1 | MH=(Sodium Chloride, Dietary OR Sodium, Dietary OR Fats, Unsaturated OR Dietary Fats OR Dietary Sucrose) |
