# Supplementary material for: The impact of food reformulation on nutrient intakes and health, a systematic review of modelling studies
Source: BMC Nutr. 2019 Jan 7;5:2. doi: 10.1186/s40795-018-0263-6 (PMC7050744; doi:10.1186/s40795-018-0263-6)
Supplement: Supplementary file 3 — Quality assessment tool. (DOCX 22 kb) [file 40795_2018_263_MOESM3_ESM.docx]

## Additional file 3 Criteria for assessment of quality of the studies

### Model design

**1) Problem, objective and scope**

Support questions:

- *Were the decision problem, modeling objective and scope clearly stated and described?*

Judgment**:**

- Low: no
- Moderate: partly
- High: yes

**2) Intervention and comparator**

Support questions:

- *Were the intervention and the comparator clearly defined and described?*
- *Were the intervention and the comparator consistent with, and adequate to the decision problem/objective to address and the policy context?*
- *Was the reformulation intervention feasible from a technical point of view? Was feasibility addressed in terms of potential reactions from the industry and/or the consumers?*

Judgment:

- Low: intervention and comparator not clearly described, feasibility not addressed
- Moderate: intervention and comparator clearly described but not consistent/adequate with/to the decision problem/objective, feasibility unclear or not discussed
- High: intervention and comparator clearly described and consistent with the objective, feasibility proved (reformulation based on existing schemes, industry and consumers behaviors taken into account)

**3) Time horizon**

Support questions:

- *Was the time horizon adequate, to comprehensively include all policy-relevant outcomes (e.g. long-term health ouctomes)?*
- *When estimating the effect of reformulation, did the model consider time trends in consumption and/or in the incidence and prevalence of modelled diseases (e.g. due to improved treatment management)?*

Judgment:

- Low: time horizon <1 year or no specific time horizon
- Moderate: time horizon <25y and consideration of time trends not clear/not used. Neither low nor high quality studies.
- High: time horizon considering cohort lifetimes or >25y, model taking into account time trends

**4) Parameters**

Support questions:

- *Were all the sources of the model parameters clearly reported in the text or in the appendix?*
- *Were the best data sources used to inform model parameters?*
- *Were the link parameters from nutrient intake to surrogate outcomes and from surrogate outcomes to clinical outcomes taken from valid and reliable studies (e.g. meta-analysis of RCTs, or large sample prospective cohort studies)?*

Judgment**:**

- Low: model parameters sources not properly reported
- Moderate: not all parameters properly reported/coming from reliable sources/adequate
- High: all model parameters clearly reported, coming from reliable sources and adequate

**5) Relevant outcomes**

Support questions:

- *Was there any important outcome missing?*

Judgment:

- Low: clinical outcomes or QALYs missing
- Moderate: only intake or surrogate outcomes
- High: none, all the relevant outcomes are reported

**6) Uncertainty**

Support questions:

- *Was uncertainty considered and properly addressed?*
- *Was uncertainty reported by means of Standard errors, sensitivity and scenario analysis, simulations results etc.?*

Judgment:

- Low: no uncertainty considered
- Moderate: only one-way and/or two way sensitivity analysis done or uncertainty estimates not reported
- High: Probabilistic sensitivity analysis done or other uncertainty measures correctly reported in accordance with the study design

### Validation

**7) Internal and external Validation**

Support questions:

- *Was the used model previously validated in other studies with respect to both internal and external validity (e.g. IMPACT CHD)?*
- *If not, were the processes to assess the external and internal validity of the model documented in detail?*

Judgment:

- Low: validation not mentioned/model not previously validated
- Moderate: validation mentioned but not clearly documented
- High: model validated in other studies/with observed data

**8) Face validity**

Support questions:

- *Does the model consider all aspects that are needed to make its results credible and realistic?*
- *Are all causal steps linking the intervention to the outcomes (e.g. uptake of the intervention by the industry, consumer reactions, changes in intake/risk factors, changes in health outcomes) properly and credibly represented and modelled*
- *Were implementation issues (e.g. stepped adherence by the industry) considered?*

Judgment**:**

- Low: no (e.g. scenario modelled not deemed realistic based on judgments on technical feasibility of the reformulation envisaged, or lack of considerations of aspects related to manufacturers and consumers behaviors)
- Moderate: Face validity aspects partly addressed. Neither low nor high quality studies.

High: yes (e.g. all aspects of the decision problem duly and credibly taken into account, reformulation scenarios are informed based on evidence or expert opinion, multiple scenario were modelled to account for the uncertain reactions of manufacturers and consumers)

### Applicability

**9) Transparency and flexibility**

Support questions:

- *Was the technical documentation sufficiently transparent and detailed to (potentially) allow for replication (e.g. made available openly or under agreements that protect intellectual property)?*

Judgment**:**

- Low: no technical documentation mentioned
- Moderate: technical documentation existing, but not available
- High: technical documentation existing and fully available
