# Supplementary material for: The impact of food reformulation on nutrient intakes and health, a systematic review of modelling studies
Source: BMC Nutr. 2019 Jan 7;5:2. doi: 10.1186/s40795-018-0263-6 (PMC7050744; doi:10.1186/s40795-018-0263-6)
Supplement: Supplementary file 4 — Additional study data. (DOC 104 kb) [file 40795_2018_263_MOESM4_ESM.doc]

## Additional file 4 – Additional data extracted from the included studies

| **Authors** | **a) Model Type b) model name (if any)** | **Target population** | **Model time horizon** | **link intake --> risk factor.  (Type of evidence)** | **link nutrient intake --> risk factor (literature source)** | **link risk factor --> clincial outcome. (Type of evidence)** | **link surrogate --> clincial 0utcome.  (literature source)** | **Funding sources** | **Assess consumers' food basket changes following the intervention** | **Technical issues with reformulation (if available)** | **Target country / region** |
| --- | --- | --- | --- | --- | --- | --- | --- | --- | --- | --- | --- |
| Cogswell et al. (2017) [1] | a) Mathematical/statistical model | Overall population | Not modelled |  |  |  |  | Public | No | Yes (reformulation based on NSRI and Health Canada standards and benchmarks) | Canada |
| Yeung et al. (2017) [2] | a) Mathematical/statistical model | Overall population | Not modelled |  |  |  |  | Not reported | No | Yes | Australia |
| Briggs et al. (2017) [3] | a) Epidemiological model (comparative risk assessment) b) PRIME | Overall population | 1y | Meta-regression of RCTs or cohort studies | estimated by the authors |  |  | Self funded | No (consumption is assumed constant) | Yes (limits of the reformulation scenario are set based on true industrial policies) | UK |
| Pearson-Stuttard (2016) [4] | a) Epidemiological model b) IMPACT_SEC CHD | Overall population | 10y | Meta-analysis of prospective cohort studies | Mozaffarian et al. (2006) (direct link between TFA and CHD) |  |  | Public | No | No, although assumptions of 100% reduction are common for industrial TFA and reflect existing policies (Bans on TFA) | UK |
| Masset et al. (2016) [5] | a) Mathematical/statistical model | Population 4-19y | Not modelled |  | NA |  |  | Private (NESTEC Sa) | No (It considers though that consumers were less likely to opt for pass pizzas which could be explained by lower preference, lower availability or higher prices) | No Nutrients' levels downsized to meet standards independently and without any technical consideration in the reformulation scenario | USA |
| Wilson et al. (2016) [6] | a) Dynamic, discrete time, deterministic Markov model b) BODE^3 | Population aged >35y | Cohort life-time | Regression of multiple observational studies | Law et al. (1991) | - IDP meta-analysis from prospective studies (Prospective Studies Collaboration) | Lewington et al (2002) | Public | No | Yes  (Reduction target informed by the UK Salt Reduction Targets for 2017 - formulated in consultation with the industry) | UK |
| Food and Drink Industry Ireland (2016) [7] | a) Mathematical/statistical model b) Creme Nutrition model | Overall population aged 1-90y | Not modelled |  | 18-90 y |  |  | Private | No | Yes (Products were reformulated for real by 14 voluntary firms) | Ireland |
| Leroy et al. (2016) [8] | a) Epidemiological model b) Dietron | Adult population (age not specified) | 1y | Dietron model | Dietron model | - meta-analysis of cohort studies, RCTs, case-control studies | - Prospective Studies Collaboration (2002, 2007,2009) - American Institute for Cancer Research (AICR) 2003 | public | No (although reformulation assumptions restricted to ranges of nutritional values already existing in the market --> acceptable by consumers) | Yes (Reformulation assumptions restricted to ranges of nutritional values already existing in the market --> feasible by producers) | France |
| Ma et al. (2016) [9] | a) Mathematical/statistical model | Adult population | 5y | mathematical modelling | Hall KD, Jordan PN. Modeling weight-loss maintenance to help prevent body weight regain | - Mathematical model (physiological adaptation to changes in bodyweight) | Hall et al. (2008) | No funding | No (Consumption of SSBs assumed constant for the whole observation period) | Yes (Feasibility of sugar reduction in SSBs was assumed. Also fruit juices were reduced in sugar content although technical issues are acknowledgeed and addressed with two different scenarios) | UK |
| Nghiem et al. (2016) [10] | a) Dynamic, discrete time, probabilistic Markov model b) BODE^3 | Overall population aged 35+ | Cohort life-time | Regression of multiple observational studies | Law et al. (1991) | - Observational data | Law et al. (1991) | public | No | Yes (Intervention sizes were informed from previous studies and existing regulations) | New Zeland |
| Choi et al. (2016) [11] | a) Discrete-time individual-level microsimulation model | Population aged 18-85 | 10y | Multicenter RCT (DASH trial) | Sacks et al. (2001) | - prospective cohort study (Framingham Heart Study) | Framingham Heart Study 2005 | public | Yes (Consider table salt addition and possible substitution of low-salt food with saltier alternatives) | Yes (all reformulation are deemed feasible) | USA |
| Allen et al. (2015) [12] | a) Epidemiological model b) IMPACT_TFA | Overall population | 6y | Meta-analysis of prospective cohort studies | Mozaffarian et al. (2006) (direct link between TFA and CHD) |  |  | Public | No | No, although assumptions of 100% reduction are common for industrial TFA and reflect existing policies (Bans on TFA) | UK |
| Gillespie et al. (2015) [13] | a) Epidemiological model b) IMPACT_SEC CHD | Population aged >25y | 10y | Meta-analysis of RCTs | He et al. (2013) | - IDP meta-analysis from prospective studies (Prospective Studies Collaboration) | Lewington et al (2002) | Public | No  (It considers though that consumers might detect reformulation and switch products or add discretionary salt) | Yes likely salt reductions elicited from expert opinions and not directly measured based on true reformulation policies (Assumes that substantial room for further salt reduction exists within the technical and safety requirements) | England |
| Bruins et al. (2015) [14] | a) Mathematical/statistical model + WHO model for DALY estimation | Adult population aged >20y | Cohort life-time | Meta-analysis of RCTs | He et al. (2013) | - meta-analysis of RCTs - prospective cohort studies | Law et al. (2009)  Lewington et al. (2002) Framingham Study (2005) | Self-funded | No (Advices for small steps changes since consumers may add table salt back) | Yes  (Feasibility of sodium reduction quantities taken from the literature) | Netherlands |
| Wilcox et al. (2015) [15] | a) Epidemiological model b) IMPACT CHD | Overall population aged >25y | 10y | Meta-analysis of RCTs | He & MacGregor (2004) | - IDP meta-analysis from prospective studies (Prospective Studies Collaboration) | Lewington et al (2002) | EU - FP project | No | yes (data taken from Smith-Spangler et al. Who did considered technical issues) | Syria |
| Hendriksen et al. (2015) [16] | a) Mathematical/statistical model | Overall population | Not modelled |  | NA | NA | NA | Public  Dutch Government | No | Yes (miminum technological fesible levels defined based on different criteria such as palatability, shelf-life, food processing techniques etc ) | Netherlands |
| Dötsch-Klerk et al. (2015) [17] | a) Mathematical/statistical model | Population 2-80y for US Population 19-64y for UK Population 18-30y for NL | Not modelled |  | NA | NA | NA | Private (Unilever) | No | Not specified/unclear (Process leading to the definition of sodium targets for foods is described, but no comments are made on technical feasibility) | UK USA Netherlands |
| Nghiem et al. (2015) [18] | a) Dynamic, discrete time, probabilistic Markov model b) BODE^3 | Overall population aged 35+ | Cohort life-time | Regression of multiple observational studies | Law et al. (1991) | - IDP meta-analysis from prospective studies (Prospective Studies Collaboration) | Lewington et al (2002) | public | No | yes, based on real legislation mandating limits on sodium levels in certain foods (South Africa, Europe). | New Zeland |
| Hendriksen et al. (2014) [19] | a) Static, discrete time, deterministic Markov model b) RIVM-CDM | Overall population adult population aged > 20 y | 20y for clinical outcomes; cohort lifetime for for DALYs | Meta-analysis of RCTs | He & MacGregor (2004) | - IDP meta-analysis from prospective studies (Prospective Studies Collaboration) - mixed effect regression model from cohort studies | Lewington et al.(2002)  Framingham study (2005) Cardiovascular Health Study (2000)  Health ABC Study (2008) EPESE East Boston Study (1999)  EPESE New Haven Study (2000)  Physicians Health Study (2009) | not reported | No | Not specified/unclear (Not specified although the paper affirms sodium reduction were not linear across foods) | Netherlands |
| Mason et al. (2014) | a) Epidemiological model b) IMPACT CHD | Adult population | 10y | Meta-analysis of RCTs | He & MacGregor (2004) | - IDP meta-analysis from prospective studies (Prospective Studies Collaboration) | Lewington et al (2002) | public | No | No | Tunisia Syria Palestine Turkey |
| Collins et al. (2014) [20] | a) epidemiological model b) IMPACT CHD | Overall population aged 25+ y | 10y | Meta-analysis of RCTs | He & MacGregor (2004) | - IDP meta-analysis from prospective studies (Prospective Studies Collaboration) | Lewington et al (2002) | public | No | Yes (reformulation effects taken from real ongoing reformulation programs) | UK |
| Konfino et al. (2013) [21] | a) Static, discrete time, deterministic Markov model b) CVD Policy Model Argentina | Population aged 35-84y | 10y | Meta-analysis of RCTs | He & MacGregor (2004) |  |  | Public | No  (Considered in the limitation though: consumers might replace with other, saltier products) | No (The model assumes a reduction in consumption by 4% a year, without directly linking such reduction to specific reformulation strategies) | Argentina |
| Bertram et al. (2012) [22] | a) Epidemiological model (comparative risk assessment) b) WHO CRA | Overall population (age not specified) | 1y | Regression of multiple observational studies | Law et al. (1991) | - IDP meta-analysis from prospective studies (Prospective Studies Collaboration) | Prospective studies collaboration 2002 | Private  (B&M gates found.) | No (Assumes no changes in other high salt foods would occur after intervention) | Yes  Magnitude of salt reduction justified based on a technical study on reformulation for bread and soups - Mentions literature that reports how other micronutrients were simultaneously increased while reducing salt | South Africa |
| Cobiac et al. (2012) [23] | a) Dynamic, discrete time, probabilistic Markov model b) ischaemic heart disease (IHD) and stroke prevention model | Overall population aged 35 - 84y | Cohort life-time | Regression of multiple observational studies | Law et al. (1991) | -meta-analysis of RCTs | Law et al. (2009) | public | No | Yes (values based on true levels observed in the tick programme) | Australia |
| Temme et al. (2011) [24] | a) Mathematical/statistical model (MonteCarlo Risk Assessment model) | Overall population | Not modelled |  |  |  |  | public | No (pre-intervention consumption was used as baseline to calculate nutrient intake changes) | Yes (nutrient content of reformulated products taken from real cases promoted by "the Dutch Task Force for the Improvement of the Fatty Acid Composition (TFIFAC)") | Netherlands |
| Combris et al. (2011) [25] | a) Mathematical/statistical model | Adult population | Not modelled |  |  |  |  | public | No (Pre-intervention consumption was used as baseline to calculate nutrient intake changes) | No (Nutrients in each food item were reformulated independently) | France |
| Hendriksen et al. (2011) [26] | a) Mathematical/statistical model | Young adults aged 19-30y | Not modelled | mathematical modelling | McNeill G (2000) Energy intake and expenditure | - Basic metabolic rate (Schofield equation) | Schofield (1985) | public | No (assumed no energy compensation occurs) | No (sugar content entirely substituted and aspects such as shelf life and palatability not considered) | Netherlands |
| Cobiac et al. (2010) [27] | a) Epidemiological model (potential impact factor) + multi-states life tables | Adult population aged 30 years and older | Cohort life-time | Regression of multiple observational studies | Law et al. (1991) | - IDP meta-analysis from prospective studies (Prospective Studies Collaboration) | Prospective studies collaboration 2002 | public | No (Behavioural response not directly measured in the intervention study, but reference to literature: consumers gradually adapt to lower salt products and reduction cannot be detected --> Consumers are unlikely to compensate for the moderate reduction in salt intake nor to change food basket) | Yes (Reformulated foods are the ones really observed in the voluntary Tick programme) | Australia |
| Smith-Spangler et al. (2010) [28] | a) Static, discrete time, probabilistic Markov model | Adult population aged 40-85 y | Cohort life-time | Multicenter RCT (DASH trial) | Sacks et al. (2001) | - IDP meta-analysis from prospective studies (Prospective Studies Collaboration) | Lewington et al (2002) | public | No (Issue somehow addressed: unintended consequences are not studied, because literature suggests no health risks are considered + appeal should remain stable to consumers + there might be positive consequences, too - reduced consumption of unhealthy components) | Yes (by definition these strategies are in collaboration with industry + mentioned that technological difficulties may limit the magnitude of sodium reduction) | USA |
| Roodenburg et al. (2009) [29] | a) Mathematical/statistical model (MonteCarlo Risk Assessment model) | Overall population aged 19-30y | Not modelled |  |  |  |  | Private (Unilever) | Yes  (a scenario is considered where the basic reformulation is corrected for the difference in energy density between the original and the replaced food --> Consumers compensate for the dicrease in energy intake by eating more replaced foods) | No | Netherlands |
| Rubinstein et al. (2009) [30] | a) Static, continuous time, deterministic Markov model + Multi-state life table  b) PopMod/WHO | Overall population | Cohort life-time | -meta-analysis of RCTs | He & McGregor (2003) | - prospective cohort study (Framingham Heart Study) | Framingham Heart Study 2005 | Program of Epidemiological Surveillance (VIGI+A). Ministry of Health | No (assumes reformulated bread is not detected as less palatable - informed by local experience) | Yes (target reformulation based on real reformulation project ongoing in Argentina) | Argentina |
| Husøy et al. (2008) [31] | a) Mathematical/statistical model | Overall population | Not modelled |  |  |  |  | public | No (energy compensation not taken into account) | No (sugar content entirely substituted and aspects such as shelf life and palatability not considered) | Norway |
| Murray et al. (2003) [32] | a) Static, continuous time, probabilistic Markov Model + Multi-state life table  b) PopMod/WHO | Overall Population | Cohort life-time | Regression of multiple observational studies | Law et al (1991),  Frost et al. (1991) | - mathematical modelling on Framingham off spring study | The World Health Report (WHO 2002) | Not mentioned | No (Only issue addressed is the fact that small reductions in salt intake are not discernible on grounds of taste so people should not resist the new foods) | No (Despite voluntary reformulation is explicitly defined as cooperation with industry for stepwise decrease in salt, study assumes a % reduction in intake without explicit considerations of feasibility) | AMR-B SEAR-D EUR-A |

References

1. Cogswell ME, Patel SM, Yuan K, Gillespie C, Juan W, Curtis CJ, et al. Modeled changes in US sodium intake from reducing sodium concentrations of commercially processed and prepared foods to meet voluntary standards established in North America: NHANES. Am J Clin Nutr. 2017;106:530–40. doi:10.3945/ajcn.116.145623.

2. Yeung CHC, Gohil P, Rangan AM, Flood VM, Arcot J, Gill TP, et al. Modelling of the impact of universal added sugar reduction through food reformulation. Sci Rep. 2017;7:17392. doi:10.1038/s41598-017-17417-8.

3. Briggs ADM, Mytton OT, Kehlbacher A, Tiffin R, Elhussein A, Rayner M, et al. Health impact assessment of the UK soft drinks industry levy: a comparative risk assessment modelling study. Lancet Public Heal. 2017;2:e15–22. doi:10.1016/S2468-2667(16)30037-8.

4. Pearson-Stuttard J, Hooton W, Critchley J, Capewell S, Collins M, Mason H, et al. Cost-effectiveness analysis of eliminating industrial and all trans fats in England and Wales: modelling study. J Public Health (Bangkok). 2016;39:574–82. doi:10.1093/pubmed/fdw095.

5. Masset G, Mathias KC, Vlassopoulos A, Mölenberg F, Lehmann U, Gibney M, et al. Modeled Dietary Impact of Pizza Reformulations in US Children and Adolescents. PLoS One. 2016;11:e0164197. doi:10.1371/journal.pone.0164197.

6. Wilson N, Nghiem N, Eyles H, Mhurchu CN, Shields E, Cobiac LJ, et al. Modeling health gains and cost savings for ten dietary salt reduction targets. Nutr J. 2016;15:44. doi:10.1186/s12937-016-0161-1.

7. Food and Drink Industry Ireland. The FDII/Creme Global Reformulation Project. 2016. http://www.fooddrinkireland.ie/Sectors/FDII/FDII.nsf/vPages/Publications~fdii-creme-global-reformulation-report/$file/The FDIICreme Global Reformulation Project.pdf.

8. Leroy P, Réquillart V, Soler L-G, Enderli G. An assessment of the potential health impacts of food reformulation. Eur J Clin Nutr. 2016;70:694–9. doi:10.1038/ejcn.2015.201.

9. Ma Y, He FJ, Yin Y, Hashem KM, MacGregor GA. Gradual reduction of sugar in soft drinks without substitution as a strategy to reduce overweight, obesity, and type 2 diabetes: a modelling study. lancet Diabetes Endocrinol. 2016;4:105–14. doi:10.1016/S2213-8587(15)00477-5.

10. Nghiem N, Blakely T, Cobiac LJ, Cleghorn CL, Wilson N. The health gains and cost savings of dietary salt reduction interventions, with equity and age distributional aspects. BMC Public Health. 2016;16:423. doi:10.1186/s12889-016-3102-1.

11. Choi SE, Brandeau ML, Basu S. Expansion of the National Salt Reduction Initiative: A Mathematical Model of Benefits and Risks of Population-Level Sodium Reduction. Med Decis Making. 2016;36:72–85. doi:10.1177/0272989X15583846.

12. Allen K, Pearson-Stuttard J, Hooton W, Diggle P, Capewell S, O’Flaherty M. Potential of trans fats policies to reduce socioeconomic inequalities in mortality from coronary heart disease in England: cost effectiveness modelling study. BMJ. 2015;351:h4583. http://www.ncbi.nlm.nih.gov/pubmed/26374614. Accessed 12 Jan 2018.

13. Gillespie DOS, Allen K, Guzman-Castillo M, Bandosz P, Moreira P, McGill R, et al. The Health Equity and Effectiveness of Policy Options to Reduce Dietary Salt Intake in England: Policy Forecast. PLoS One. 2015;10:e0127927. doi:10.1371/journal.pone.0127927.

14. Bruins MJ, Dötsch-Klerk M, Matthee J, Kearney M, van Elk K, Weber P, et al. A Modelling Approach to Estimate the Impact of Sodium Reduction in Soups on Cardiovascular Health in the Netherlands. Nutrients. 2015;7:8010–9. doi:10.3390/nu7095375.

15. Wilcox ML, Mason H, Fouad FM, Rastam S, al Ali R, Page TF, et al. Cost-effectiveness analysis of salt reduction policies to reduce coronary heart disease in Syria, 2010-2020. Int J Public Health. 2015;60 Suppl 1:S23-30. doi:10.1007/s00038-014-0577-3.

16. Hendriksen MAH, Verkaik-Kloosterman J, Noort MW, van Raaij JMA. Nutritional impact of sodium reduction strategies on sodium intake from processed foods. Eur J Clin Nutr. 2015;69:805–10. doi:10.1038/ejcn.2015.15.

17. Dötsch-Klerk M, Goossens WPMM, Meijer GW, van het Hof KH. Reducing salt in food; setting product-specific criteria aiming at a salt intake of 5 g per day. Eur J Clin Nutr. 2015;69:799–804. doi:10.1038/ejcn.2015.5.

18. Nghiem N, Blakely T, Cobiac LJ, Pearson AL, Wilson N. Health and economic impacts of eight different dietary salt reduction interventions. PLoS One. 2015;10:e0123915. doi:10.1371/journal.pone.0123915.

19. Hendriksen MA, Hoogenveen RT, Hoekstra J, Geleijnse JM, Boshuizen HC, van Raaij JM. Potential effect of salt reduction in processed foods on health. Am J Clin Nutr. 2014;99:446–53. doi:10.3945/ajcn.113.062018.

20. Collins M, Mason H, O’Flaherty M, Guzman-Castillo M, Critchley J, Capewell S. An economic evaluation of salt reduction policies to reduce coronary heart disease in England: a policy modeling study. Value Health. 2014;17:517–24. doi:10.1016/j.jval.2014.03.1722.

21. Konfino J, Mekonnen TA, Coxson PG, Ferrante D, Bibbins-Domingo K. Projected Impact of a Sodium Consumption Reduction Initiative in Argentina: An Analysis from the CVD Policy Model – Argentina. PLoS One. 2013;8:e73824. doi:10.1371/journal.pone.0073824.

22. Bertram MY, Steyn K, Wentzel-Viljoen E, Tollman S, Hofman KJ. Reducing the sodium content of high-salt foods: effect on cardiovascular disease in South Africa. S Afr Med J. 2012;102:743–5. http://www.ncbi.nlm.nih.gov/pubmed/22958695. Accessed 29 Apr 2017.

23. Cobiac LJ, Magnus A, Lim S, Barendregt JJ, Carter R, Vos T. Which interventions offer best value for money in primary prevention of cardiovascular disease? PLoS One. 2012;7:e41842. doi:10.1371/journal.pone.0041842.

24. Temme EHM, Millenaar IL, Van Donkersgoed G, Westenbrink S. Impact of fatty acid food reformulations on intake of Dutch young adults. Acta Cardiol. 2011;66:721–8.

25. Combris P, Goglia R, Henini M, Soler LG, Spiteri M. Improvement of the nutritional quality of foods as a public health tool. Public Health. 2011;125:717–24. doi:10.1016/j.puhe.2011.07.004.

26. Hendriksen MA, Tijhuis MJ, Fransen HP, Verhagen H, Hoekstra J. Impact of substituting added sugar in carbonated soft drinks by intense sweeteners in young adults in the Netherlands: example of a benefit–risk approach. Eur J Nutr. 2011;50:41–51. doi:10.1007/s00394-010-0113-z.

27. Cobiac LJ, Vos T, Veerman JL. Cost-effectiveness of interventions to reduce dietary salt intake. Heart. 2010;96:1920–5. doi:10.1136/hrt.2010.199240.

28. Smith-Spangler CM, Juusola JL, Enns EA, Owens DK, Garber AM. Population Strategies to Decrease Sodium Intake and the Burden of Cardiovascular Disease. Ann Intern Med. 2010;152:481. doi:10.7326/0003-4819-152-8-201004200-00212.

29. Roodenburg AJC, Temme EHM, Davies OH, Seidell JC. Potential impact of the Choices Programme on nutrient intakes in the Dutch population. Nutr Bull. 2009;34:318–23. doi:10.1111/j.1467-3010.2009.01767.x.

30. Rubinstein A, García Martí S, Souto A, Ferrante D, Augustovski F. Generalized cost-effectiveness analysis of a package of interventions to reduce cardiovascular disease in Buenos Aires, Argentina. Cost Eff Resour Alloc. 2009;7:10. doi:10.1186/1478-7547-7-10.

31. Husøy T, Mangschou B, Fotland TØ, Kolset SO, Nøtvik Jakobsen H, Tømmerberg I, et al. Reducing added sugar intake in Norway by replacing sugar sweetened beverages with beverages containing intense sweeteners - a risk benefit assessment. Food Chem Toxicol. 2008;46:3099–105. doi:10.1016/j.fct.2008.06.013.

32. Murray CJ, Lauer JA, Hutubessy RC, Niessen L, Tomijima N, Rodgers A, et al. Effectiveness and costs of interventions to lower systolic blood pressure and cholesterol: a global and regional analysis on reduction of cardiovascular-disease risk. Lancet. 2003;361:717–25. doi:10.1016/S0140-6736(03)12655-4.
